# Robust adaptive control with dung beetle optimization algorithm and disturbance observer for load displacement tracking of shock absorber damper test bench

**DOI:** 10.1371/journal.pone.0314775

**Published:** 2025-02-24

**Authors:** Xiangfei Tao, Kailei Liu, Dong Han, Jing Yang, Hongbin Qiang

**Affiliations:** 1 School of Mechanical Engineering, Jiangsu University of Technology, Changzhou, Jiangsu, China; 2 School of Mechanical Engineering, Zhejiang University, Hangzhou, Zhejiang, China; Imperial College London, UNITED KINGDOM OF GREAT BRITAIN AND NORTHERN IRELAND

## Abstract

Shock absorbers are indispensable components utilized for vibration mitigation in various fields, including construction, bridges, wind power, and pipelines. The shock absorber test bench serves as an essential apparatus for evaluating the dynamic and static characteristics of these absorbers. To address the significant tracking errors between actual and expected displacements during displacement loading on shock absorber damper test benches, a novel control strategy is proposed. This strategy incorporates a robust adaptive controller (RAC) enhanced by a dung beetle optimization (DBO) algorithm and a disturbance observer (DO). The dung beetle optimization algorithm is specifically designed to iteratively optimize the control parameters of the robust adaptive controller. Concurrently, a disturbance observer is implemented to accurately estimate external disturbances and perform feedforward compensation. A mathematical model of the electro-hydraulic servo control system of the test bench is established, and the stability of the proposed controller is rigorously verified using Lyapunov theory. To simulate and analyze the control method for the electro-hydraulic servo system of the test bench, a joint simulation model integrating Simulink and AEMSim is constructed. The performance of the proposed robust adaptive controller with DBO and DO is compared against an unoptimized robust adaptive controller and a traditional PID controller in terms of load displacement tracking. Simulation results demonstrate that the control method proposed in this study significantly outperforms other controllers in enhancing position tracking accuracy and improving system robustness. Furthermore, experimental verification was carried out on the proposed control strategy, compared with unoptimized robust adaptive control, the maximum displacement tracking error was reduced by 54.8%, and the response speed was improved by 36.3%, compared with traditional PID control, the minimum displacement tracking error was reduced by 67.4%, and the response speed was improved by 47.7%. The results confirmed the superior performance of the proposed control method.

## 1 Introduction

Shock absorbers and dampers are essential components extensively used in diverse industrial sectors, including automotive, construction, aerospace, and mechanical manufacturing. The performance of these components significantly influences the stability, safety, and longevity of the systems in which they are incorporated [1, 2]. By absorbing and dissipating vibration energy, shock absorbers effectively mitigate the vibration and impact on structures subjected to dynamic loads, thereby safeguarding the structures from potential damage and enhancing the overall safety and comfort of the system [3, 4]. Consequently, precise evaluation and testing of shock absorber and damper performance are of paramount importance. The shock absorber damper test bench is employed to assess the dynamic and static characteristics of shock absorber dampers through displacement loading, ensuring compliance with equipment requirements. Electro-hydraulic servo control technology is typically utilized in the control of shock absorber damper test benches. However, the electro-hydraulic servo displacement control system exhibits nonlinear characteristics and control parameter uncertainties, rendering accurate mathematical modeling of the system challenging. As a result, achieving high-precision control of the shock absorber damper test bench is a formidable task [5].

In recent years, a plethora of nonlinear and parameter uncertain control problems have emerged across various domains [6]. Consequently, addressing the nonlinearity, parameter uncertainty, and unknown disturbances inherent in diverse control systems has become a focal point of research for scholars both domestically and internationally. Traditional nonlinear control theory employs various linearization techniques to transform nonlinear systems into linear systems under specific operating conditions [7]. For example, Taylor expansion linearization is achieved by performing Taylor expansion near a specific operating point (equilibrium point or operating point) of the nonlinear system. This method retains the first-order term (and sometimes the constant term), while disregarding higher-order terms, to obtain a linear approximation model [8]. Feedback linearization involves the introduction of feedback control to transform the dynamic characteristics of a nonlinear system into those of a linear system. This linearization approach necessitates the design of appropriate feedback control laws to linearize the input and output states of the system, enabling precise control using conventional linear control algorithms [9]. Segmented linearization divides the domain of a nonlinear function into multiple small intervals, approximating the original nonlinear function with a linear function within each interval. By concatenating these linear segments, a piecewise linear approximation model over the entire domain can be obtained [10]. To tackle the problem of nonlinear uncertain parameters in electro-hydraulic servo systems, an adaptive backstepping control method utilizing an integral type Lyapunov function has been proposed. This method initially defines an integral type Lyapunov function to transform the nonlinear uncertain parameters in the electro-hydraulic servo system into linear representations [11].

PID control, the most classical linear control algorithm, is extensively employed across various industrial production domains. However, electro-hydraulic servo systems, being typical nonlinear systems, are significantly affected by parameter uncertainty, which can severely interfere with system performance. Consequently, traditional PID control algorithms struggle to achieve the requisite control accuracy and stability for these systems [12]. In response to the nonlinearity and uncertain control parameters inherent in electro-hydraulic servo systems, adaptive control algorithms have been proposed. These algorithms automatically identify changes in the system and its operational environment, facilitating corresponding adjustments in control strategies. This adaptability ensures system stability control even after certain parameters have been altered [13]. Adaptive control represents a significant advancement in nonlinear control, with such algorithms demonstrating excellent suppression of system uncertainty. As a result, these algorithms are widely implemented in nonlinear time-varying systems, including hydraulic servo systems [14, 15]. In scenarios where hydraulic systems exhibit a certain range of parameter uncertainty and lack precise mathematical modeling, robust control strategies can be employed to ensure good dynamic stability performance of the closed-loop control system [16]. A sign integral robust controller based on nonlinear adaptive control theory was proposed in [17]. This controller addresses the issues of nonlinear friction and position nonlinear interference in servo hydraulic systems, thereby enhancing the control accuracy of electro-hydraulic servo systems. In [18], a nonlinear adaptive control method for hydraulic systems with nonlinear parameters was introduced. This method compensates for uncertain nonlinear parameters caused by changes in the current control volume through a nonlinear adaptive controller with adaptive laws. A new Lyapunov function is constructed to design an asymptotically stable adaptive controller. Furthermore, by integrating simple robust control methods, a controller and adaptive law for the servo hydraulic system are provided, capable of compensating for the uncertainty and nonlinearity of all unknown parameters in the system. An optimal robust adaptive fuzzy backstepping control algorithm for the position control of hydraulic servo systems with structured and unstructured uncertainties was proposed in [19]. This algorithm optimizes control coefficients and membership functions. Experimental results demonstrate that this control algorithm effectively suppresses chattering problems while maintaining tracking performance. In [20], an adaptive sliding mode controller based on vector field, and successfully extended the vector field guidance law from simple trajectories to different curves. Two optimization algorithms, IPSO and PGWO, were introduced to optimize the sliding mode controller and output the optimal direction angle and velocity. In [21], a composite control strategy for NFTSMC based on NRL and NFTSMDO. The design of a non singular fast terminal sliding surface ensures the convergence of the system state within a finite time and solves the singularity problem in the terminal sliding surface. A control method based on machine learning and sliding mode control was proposed in [22], using neural networks as control compensators to estimate and eliminate the dead zone of actuators, and robust sliding mode compensators as auxiliary controllers to ensure the stability and robustness of the system under model uncertainty, approximation error, and friction conditions. In [23], a novel synovial observer that solves the problem of low observation accuracy, and based on this, designed a new approach rate to enhance the stability of the system. An innovative model free self-organizing weight adaptive method was proposed in [24], which enhances the robustness of the linear quadratic regulator of the inverted pendulum electromechanical system to disturbances and parameter uncertainties, improves the design flexibility of the controller, and enhances the system’s anti-interference ability. In [25], an adaptive backstepping controller based on neural networks was proposed to model uncertainty and external disturbance phenomena in electro-hydraulic servo systems. A radial basis function neural network was constructed to approximate system uncertainty, and adaptive laws were employed to estimate control parameters. Backstepping control was utilized to eliminate nonlinear terms in the system. A high-performance adaptive controller for a specific type of electro-hydraulic system vibration table was proposed in [26]. This controller addresses issues such as nonlinearity, dynamic characteristic changes, and unexpected external disturbances. The method first establishes a nonlinear model of the elastohydrodynamic lubrication system and then proposes a control method based on a Lyapunov enhanced parameter update law through backstepping design. The stability of the control algorithm is demonstrated through Lyapunov analysis. While the aforementioned control methods represent significant advancements, there remain challenges such as complex controller algorithms and inadequate consideration of the time-varying characteristics of control parameters. These limitations continue to impact the overall control performance of hydraulic systems.

Optimization problems in numerous engineering projects have garnered extensive attention and grown increasingly complex. As a crucial research direction in engineering and computing fields, optimization problems present significant challenges in current research [27]. In complex practical optimization scenarios, traditional mathematical methods often encounter time-consuming computations and local optimization issues. However, swarm intelligence and evolutionary algorithms have made substantial progress in addressing optimization problems in engineering applications and computational science, circumventing the aforementioned drawbacks of traditional optimization methods. Among intelligent optimization algorithms, the beetle algorithm has emerged as a notable contender. Proposed in late 2022 [28], the dung beetle optimization algorithm represents a novel and efficient swarm intelligence optimization technique. This algorithm demonstrates significant advantages over currently popular methods such as particle swarm optimization, grey wolf optimization, sparrow search algorithm, sine cosine optimization, and multiverse optimization in terms of convergence speed, solution accuracy, and stability [29, 30]. The dung beetle optimization algorithm is characterized by its fewer parameters, rapid global search capabilities, has good adaptability to high-dimensional problems and easy to expand [31, 32].

Electro-hydraulic servo control systems are inherently subject to mismatched disturbances. These disturbances, which do not satisfy matching conditions, infiltrate the control system through alternative channels, thereby compromising system stability and affecting overall robustness. Consequently, active anti-interference control assumes paramount importance in these systems. Nonlinear disturbance observers have been demonstrated to effectively estimate these disturbances, enabling feedforward compensation in the controller. This approach significantly mitigates the impact of non-matching disturbances on the system. To enhance the position tracking accuracy of projectile coordination arm electro-hydraulic position servo systems, a combination of disturbance observer and backstepping control has been implemented. This integration effectively compensates for disturbances and augments system robustness [33]. Furthermore, to minimize the impact of unknown disturbances on electro-hydraulic servo systems and ensure optimal performance, disturbance observers have been constructed. These observers facilitate real-time online evaluation and estimation of internal and external dynamic disturbances within control systems [34].

The current advanced control strategies for electro-hydraulic servo systems exhibit limitations, including complex control algorithms, time-varying key parameters, and insufficient consideration of control signal disturbances. These factors contribute to suboptimal controller performance. The loading system of shock absorber damper test benches represents a typical electro-hydraulic servo control system characterized by parameter uncertainty and uncertain nonlinearity, necessitating the implementation of a robust adaptive control strategy. The application of robust adaptive control in electro-hydraulic servo systems has demonstrated unique originality and novelty. This control method innovatively solves the uncertainty problems faced by electro-hydraulic servo systems, such as parameter changes and external disturbances, by combining the advantages of adaptive control and robust control. It can not only adjust controller parameters in real-time to cope with changes in system dynamic characteristics, but also maintain robustness to uncertainties, thereby significantly improving system stability and control accuracy.

In response to the problems of nonlinearity, parameter uncertainty, and unknown external disturbances in the electro-hydraulic servo control system of the test bench, this paper proposes a robust adaptive electro-hydraulic servo position control method based on the dung beetle algorithm and disturbance observer on the basis of Lyapunov theory. The control accuracy of the controlled system has been significantly improved, and the system also demonstrates accurate estimation of uncertain parameters and flexible response to load disturbances. This article also designs a disturbance observer and a dung beetle optimization algorithm. The disturbance observer is used to accurately estimate external load disturbances and perform feedforward compensation, solving the problem of traditional robust adaptive control relying on high gain feedback to handle disturbances. The dung beetle optimization algorithm is used to iteratively optimize control parameters, solving the problem of uncertain unknown parameters in traditional robust adaptive control. This control method combines the disturbance suppression capability of the disturbance observer and the global optimization capability of the dung beetle optimization algorithm, which can significantly improve the control performance and stability of nonlinear systems. The high-precision control of displacement loading on the shock absorber damper test bench was ultimately achieved, laying a strong foundation for the dynamic and static characteristic testing of shock absorbers.

The primary contributions of this research are summarized as follows:

1. Apply robust adaptive control to nonlinear electro-hydraulic servo position control systems. And the dung beetle optimization algorithm and disturbance observer have been introduced into the traditional robust adaptive controller, thereby improving the tracking accuracy and robustness of the electro-hydraulic servo position control system.
2. Using the dung beetle optimization algorithm to approximate the uncertain parameters of the robust adaptive controller online, the uncertain parameters of the controller were optimized and estimated, and then incorporated into the controller for control.
3. Establish an interference observer to accurately estimate external load disturbances, and perform feedforward compensation on the interference information estimated online by the system, solving the problem of traditional robust adaptive control relying on high gain feedback to handle disturbances.

The remaining structure of this article is organized as follows: Section 2 presents the design scheme of the test bench. Section 3 elucidates the mathematical modeling of the hydraulic servo system on the experimental platform. Section 4 delineates the design and stability analysis of robust adaptive controllers. Section 5 compares the control performance through simulation. Section 6 evaluates the experimental control performance. Section 7 provides a comprehensive summary of the entire study.

## 2 Brief description of test bench design scheme

**Fig 1:**
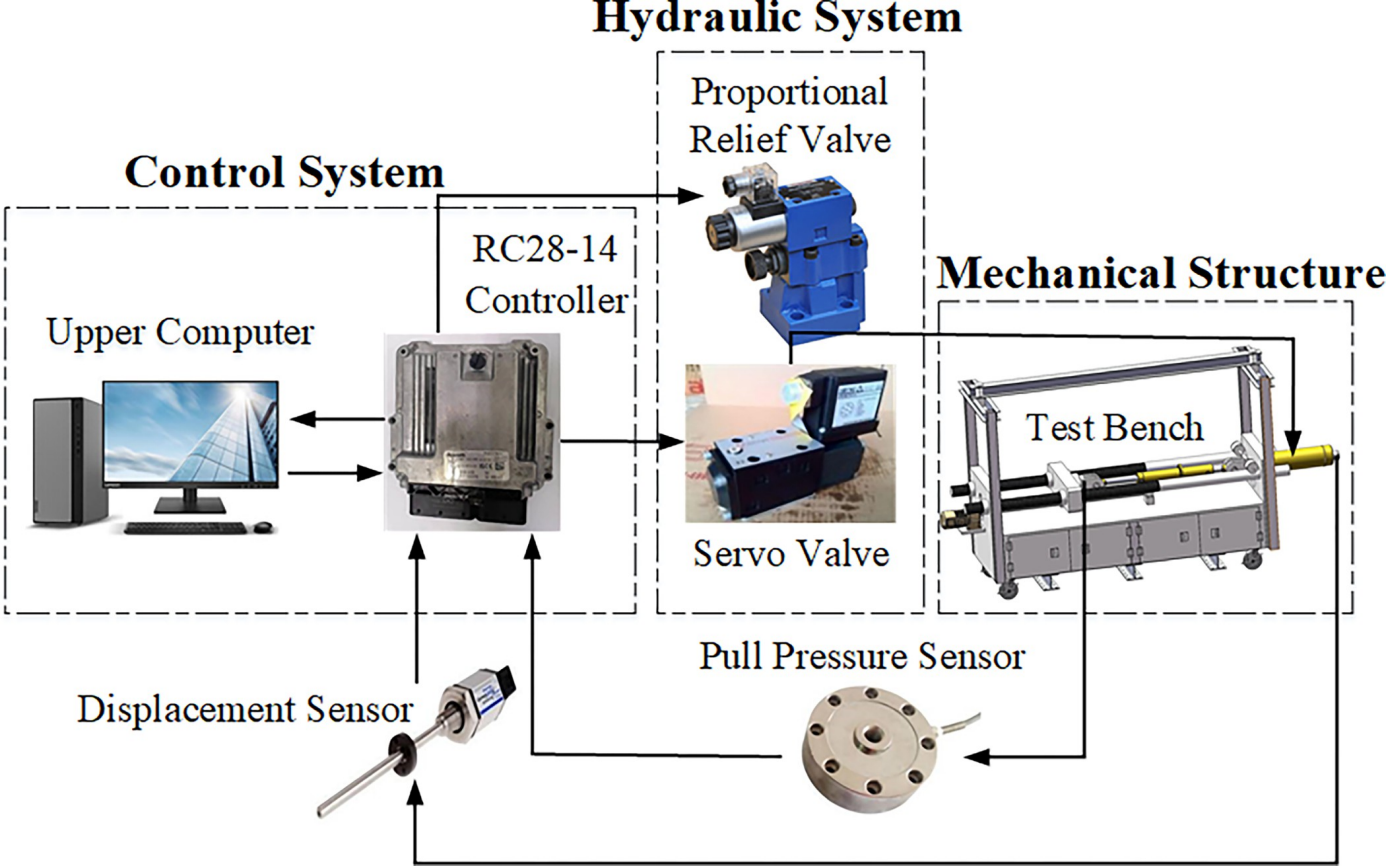
Design scheme of shock absorber test bench.

The shock absorber damper test bench design, illustrated in Fig 1, comprises a control system, hydraulic system, test bench, and auxiliary components. The shock absorber damper is mounted on the test bench, where the hydraulic cylinder piston rod generates axial displacement to tension and compress the damper for parameter testing. A displacement sensor is connected to the loading cylinder to monitor the actual loading displacement and transmit the displacement data to the RC28-14 controller. The Controller Area Network(CAN) signal of the sensor is transmitted to the upper computer through PeakCAN(instrument for downloading controller programs and communicating with the upper computer), forming a displacement closed-loop control system in Simulink. The control algorithm written in Simulink is used to correct the control signal, and then the control signal is transmitted to the RC28-14 controller through PeakCAN to control the servo valve, achieving tracking control between the actual loading displacement of the cylinder and the target loading displacement. A tension and pressure sensor, installed on the test bench’s installation head away from the loading cylinder, monitors the damper’s damping force.

The control system of the shock absorber damper test bench includes an industrial computer, RC28-14 controller, PeakCAN, DEWESoft data acquisition instrument, and Simulink control algorithm. The test interface and measurement and control algorithm are written using Simulink in Matlab software.The Simulink control algorithm primarily executes tasks such as loading parameter settings, control output, data acquisition, and processing. Post-experiment, the relationships between actual loading displacement, damping force, and time can be analyzed via a Industrial computer to derive parameters including fatigue frequency, damping index, and energy dissipation rate of the shock absorber damper.

## 3 Mathematical modeling of hydraulic servo system for test bench

**Fig 2:**
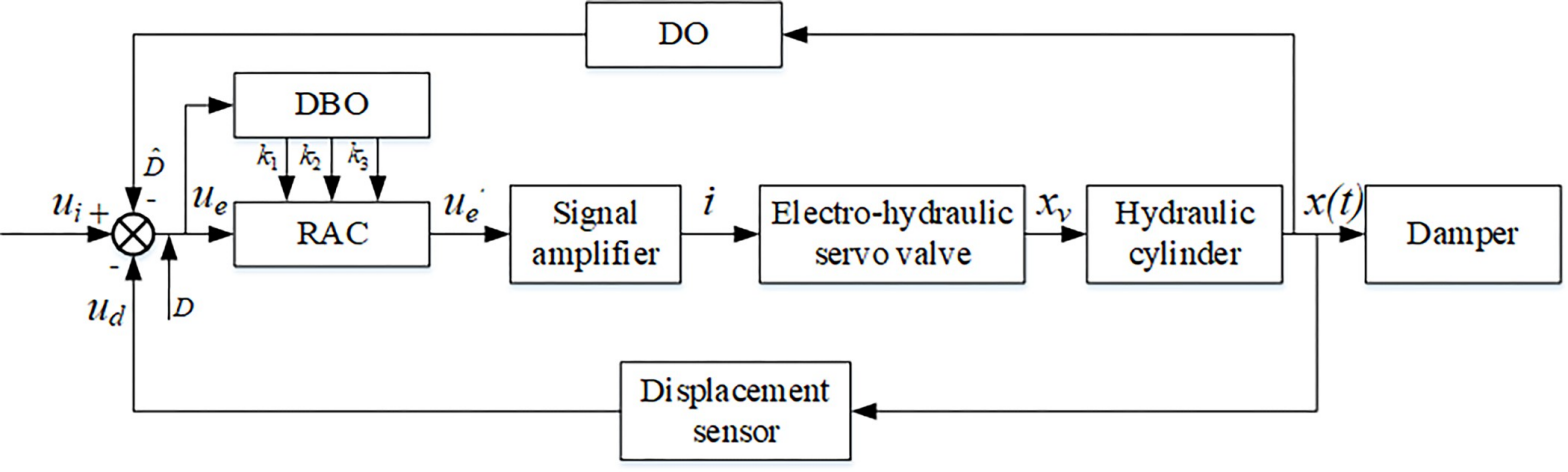
Schematic diagram of hydraulic servo displacement control system structure.

Fig 2 depicts the schematic diagram of the hydraulic servo displacement control system structure. Here, *u*_*i*_ represents the target displacement signal, *x*(t) denotes the actual output displacement of the hydraulic cylinder loaded on the test bench, and *u*_*d*_ signifies the displacement feedback signal, *D* is interference, *k*_1_, *k*_2_, *k*_3_ are control parameters.

The signal amplifier employed in this test bench is the Atros E-RI-TEB-N type. It receives a voltage control signal as input and outputs a servo valve current control signal. The amplification process is characterized by a proportional link, with its transfer function expressed as:

$$\frac{I(s)}{U(s)}=K_{a}$$ (1)

Where, *s* represents the Laplace operator, and *I*(*s*) is the Laplace transform of the amplifier output current signal; *U*(s) is the Laplace transform formula for the input voltage control signal of the amplifier, and *K*_*a*_ is the amplifier gain. In this test bench, the servo valve current input signal ranges from 4–20 mA, while the amplifier input voltage control signal spans ±10 V. Therefore, *K*_*a*_ = 0.8mA/V.

The selected displacement sensor is a Baruff magnetic displacement sensor (model BTL BNCA00-0500-B15AE420-000S92), used in conjunction with a Vidias 4–20 mA to ±10 V signal isolator. This configuration converts the displacement into a voltage signal that is readily collected by the controller. The sensor’s rapid response allows it to be modeled as a proportional component within the system. Its transfer function can be expressed as:

$$\frac{U_{i}(s)}{X_{p}(s)}=K_{i}$$ (2)

Where, *s* represents the Laplace operator, and *U*_*i*_(*s*) is the Laplace transform formula for the output voltage signal; *X*_*p*_(*s*) is the Laplace transform formula for the input displacement value signal; *K*_*i*_ is the displacement sensor gain. The signal isolator in this test bench converts the output to a 0-10V range, corresponding to a displacement range of 0-50cm. Therefore, *K*_*i*_ = 0.2V/cm.

The electro-hydraulic servo valve utilized is the Atos DLKZOR-TEB-SN-NP-L73 model. Due to its complex structure integrating mechanical, electrical, and hydraulic technologies, the input-output relationship exhibits nonlinearity, complicating accurate modeling. Consequently, this component is simplified in the current study. The displacement *x*_*v*_ of the servo valve spool and the input current signal *u* are approximated as having a proportional relationship, with the transfer function:

$$\frac{X_{v}(s)}{u(s)}=K_{sv}$$ (3)

Where, *K*_*sv*_ = *x*_*v*_*(max*)/*u*_*n*_ and *x*_*v*_*(max*) represent the maximum displacement of the servo valve core, while *u*_*n*_ represents the rated current of the electro-hydraulic servo valve, and *s* represents the Laplace operator; *X*_*v*_(*s*) represents the Laplace transform for the displacement of the slide valve; *u*(*s*) represents the Laplace transform for output flow rate; *K*_*sv*_ represents the gain with current as input and spool displacement as output. The servo valve employed in this test bench utilizes a current input signal of 4–20 mA. The valve core is positioned neutrally at an input current of 12 mA, establishing the rated current of the servo valve as *u*_*n*_ = 8mA. The maximum displacement of the servo valve spool is measured at *x*_*v*(*max*)_ = 1.5×10^−2^*m*. By substituting the rated current and maximum displacement of the valve core, the gain of valve core displacement input current is calculated as *K*_*sv*_ = 0.1875m/A.

**Fig 3:**
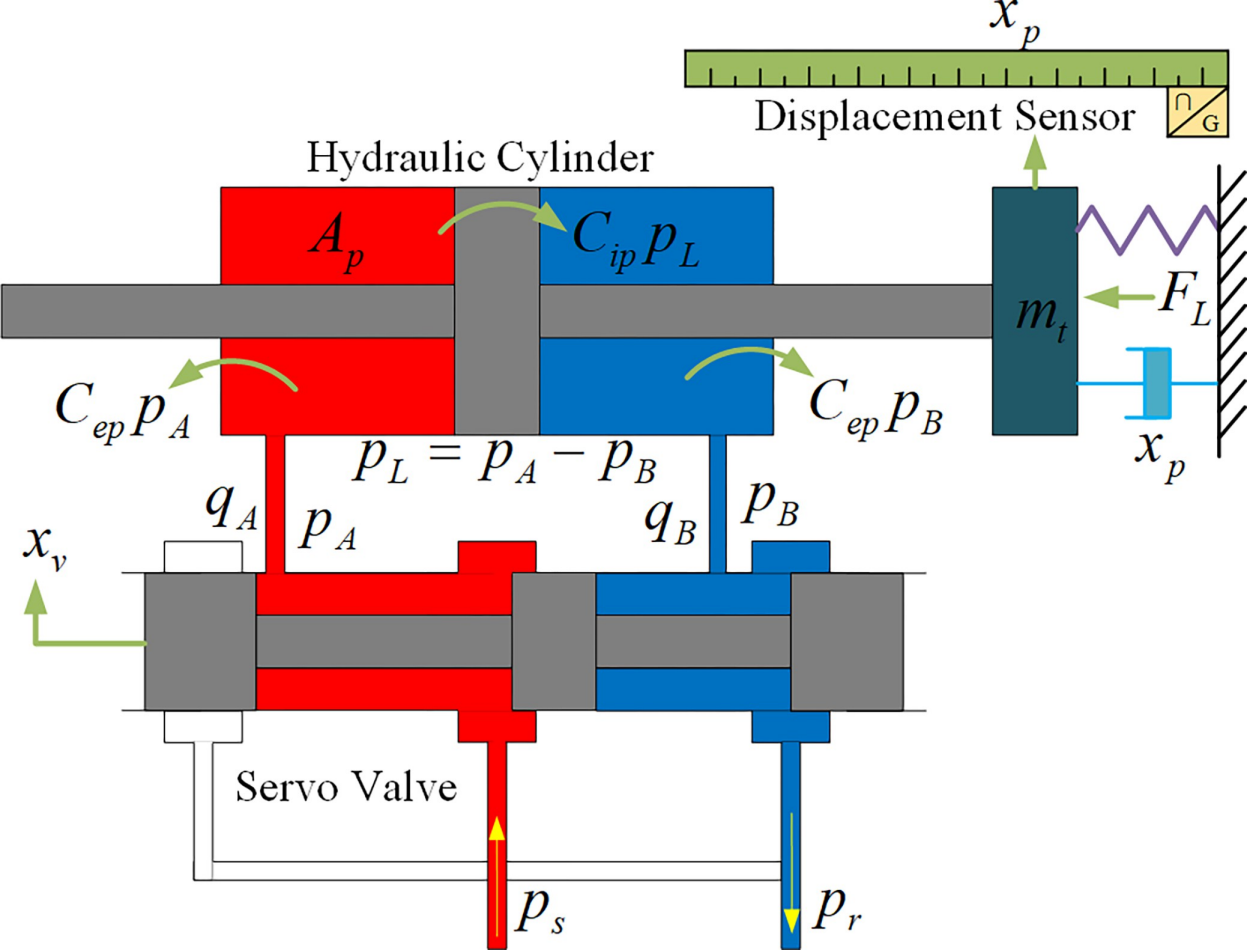
Working principle diagram of valve controlled symmetrical hydraulic cylinder.

Fig 3 illustrates the working principle diagram of the servo valve-controlled symmetrical hydraulic cylinder in the displacement loading system of the test bench. In the figure, *A*_*p*_ represents the effective pressure area of the hydraulic cylinder; *V*_*A*_ represents the effective volume of the left chamber of the hydraulic cylinder, and *V*_*B*_ represents the effective volume of the right chamber of the hydraulic cylinder; *C*_*ip*_, *C*_*ep*_ represents the leakage coefficients inside and outside the hydraulic cylinder, respectively; *p*_*A*_, *q*_*A*_ represents the left chamber pressure and flow rate of the hydraulic cylinder; *p*_*B*_, *q*_*B*_ represents the right chamber pressure and flow rate of the hydraulic cylinder; *m*_*t*_ represents the total mass acting on the piston rod, including the mass of the piston rod and the mass of the inertial load, etc; *F*_*L*_ represents the external load force; *x*_*v*_ represents the displacement of the slide valve; *x*_*p*_ represents the displacement value of the hydraulic cylinder piston rod; *p*_*s*_ represents the supply pressure, and *p*_*r*_ represents the return pressure; Let *p*_*L*_ be the load pressure, *p*_*L*_ = *p*_*A*_−*p*_*B*_; let the load flow rate be *q*_*L*_, assuming *q*_*L*_ = (*q*_*A*_−*q*_*B*_)/2.

The linear equation for the flow rate of a slide valve is expressed as:

$$q_{L}=k_{q}x_{v}\sqrt{p_{s}-sgn(x_{v})p_{L}}$$ (4)

Where, sgn(*x*) represents a sign function, and its expression is defined as:

$$sgn(x)=\left\{ \begin{array}{c} 1,x>0\\ -1,x\leq 0 \end{array}\right.$$ (5)

*k*_*q*_ represents the flow gain, and its expression is expressed as:

$$k_{q}=C_{d}\omega \sqrt{1/\rho }$$ (6)

In the formula, *C*_*d*_ represents the flow coefficient, *ω* denotes the gradient of the servo valve spool area, and *ρ* signifies the hydraulic oil density.

Substituting Eq (3) into Eq (4) yields:

$$q_{L}=k_{1}u\sqrt{p_{s}-sgn(x_{v})p_{L}}$$ (7)

Where, *k*_1_ = *k*_*q*_*k*_*sv*_ represents the total flow gain of the system.

The flow continuity equation of the hydraulic cylinder is given by:

$$q_{L}=A_{p}\frac{dx_{p}}{dt}+C_{tp}p_{L}+\frac{V_{t}}{4\beta_{e}}\times \frac{dp_{L}}{dt}+q_{n}$$ (8)

Where, *β*_*e*_ denotes the equivalent bulk modulus of elasticity of hydraulic oil, *q*_*n*_ represents a constant modeling error as a function of time; *V*_*t*_ = *V*_*A*_+*V*_*B*_ signifies the total compressed volume of the hydraulic cylinder, and *C*_*ip*_ = *C*_*ip*_+*C*_*ep*_/2 denotes the total leakage coefficient of the hydraulic cylinder.

The balance equation between the output force of the hydraulic cylinder and the load forces is expressed as:

$$A_{p}p_{L}=m_{t}\frac{d^{2}x_{p}}{dt^{2}}+B_{p}\frac{dx_{p}}{dt}+f$$ (9)

Where, *f* represents the unmodeled disturbance, which is a function of the displacement, velocity, and time of the hydraulic cylinder piston rod; *B*_*p*_ denotes the total viscous damping coefficient, encompassing hydraulic oil damping and load damping; *K* signifies the equivalent spring stiffness of elastic load; *F*_*L*_ represents the external load force.

The state variable is selected as $x=(x_{1},x_{2},x_{3})=(x_{p},{\dot{x}}_{p},Ap_{L}/m_{t})$.

The entire system can be represented in the following state space form:

$$\left\{ \begin{array}{c} {\dot{x}}_{1}=x_{2}\\ {\dot{x}}_{2}=x_{3}-a_{1}x_{2}-d(x,t)\\ {\dot{x}}_{3}=a_{2}gu-a_{3}x_{2}-a_{4}x_{3}-a_{5} \end{array}\right.$$ (10)

Where, *a* = (*a*_1_, *a*_2_, *a*_3_, *a*_4_, *a*_5_) denotes a set of unknown parameters, with $a_{1}=B_{p}/m_{t},\;a_{2}=4\beta_{e}k_{t}/m_{t}V_{t},\;a_{3}=4\beta_{e}A_{p}/m_{t}V_{t},\;a_{4}=4\beta_{e}{AC}_{tp}/m_{t}V_{t},\;a_{5}=4\beta_{e}q_{n}/V_{t},\;d(x,t)=f/m_{t},\;g=\sqrt{p_{s}-sgn(u)p_{L}}$.

Where, all unknown parameters possess known upper and lower bounds, with their lower bounds exceeding 0. The upper and lower bounds of *d*(*x*, *t*) and its first derivative are also known.

## 4 Design and stability analysis of robust adaptive controller

### 4.1 Robust adaptive law design of projection correction method

$\hat{a}$ denotes the parameter estimation of uncertain parameter *a*, while $\tilde{a}$ denotes the estimation error. The relationship between these variables is expressed as:

$$\tilde{a}=\hat{a}-a$$ (11)

The robust adaptive law is formulated as:

$$\dot{\hat{a}}={Proj}_{\hat{a}}(\Gamma \tau )$$ (12)

In the formula: Γ represents the adaptive rate diagonal positive definite matrix; τ denotes an adaptive function; ${Proj}_{\hat{a}}(\circ )$ signifies a discrete projection function, which is defined as:

$${Proj}_{\hat{a}}(\circ )=\left\{ \begin{array}{c} 0,\hat{a}={\hat{a}}_{min}and\circ <0\\ 0,\hat{a}={\hat{a}}_{max}and\circ >0\\ \circ ,other \end{array}\right.$$ (13)

Therefore, for any adaptive function τ, the following properties are observed:

$$\left\{ \begin{array}{c} \hat{a}\in \Omega =\{\hat{a}:a_{min}\leq a\leq a_{max}\}\\ {\tilde{a}}^{T}[\Gamma^{-1}{Proj}_{\hat{a}}(\Gamma \tau )-\tau ]\leq 0 \end{array}\right.$$ (14)

### 4.2 Design of robust adaptive controller

The following variables are defined as:

$$e_{1}=x_{1}-x_{1d}$$ (15)

$$e_{2}={\dot{e}}_{1}+k_{1}e_{1}=x_{2}-\alpha_{1}$$ (16)

$$e_{3}=x_{3}-\alpha_{2}$$ (17)

$$\alpha_{1}={\dot{x}}_{1d}-k_{1}e_{1}$$ (18)

In these equations: *x*_1*d*_ represents the target displacement; *α*_1_ *α*_2_ denotes the virtual control rate; *k*_1_ denotes the virtual control rate, while $G(s)=e_{1}(s)/e_{2}(s)=1/\left(s+k_{1}\right)$. It can be deduced that when *e*_2_→0 is present, there must be *e*_1_→0. Therefore, the design objective can be set to make *e*_2_ approach 0.

The Lyapunov function is constructed as follows:

$$L_{2}=e_{2}^{2}/2$$ (19)

By taking the derivative of *e*_2_ over time and substituting it into Eq (10), we can obtain:

$${\dot{e}}_{2}=x_{3}-a_{1n}x_{2}-{\dot{\alpha }}_{1}-D(x,t)$$ (20)

In the equation, $D(x,t)=(a_{2}-a_{2n})x_{2}-d(x,t)$ represents interference.

Differentiating Eq (19) yields:

$${\dot{L}}_{2}=e_{2}{\dot{e}}_{2}=e_{2}[e_{3}+\alpha_{2}-a_{1n}x_{2}-{\dot{\alpha }}_{1}-D(x,t)]$$ (21)

Thus, the virtual control rate *α*_2_ can be designed as follows:

$$\left\{ \begin{array}{c} \alpha_{2}=\alpha_{2a}+\alpha_{2s}\\ \alpha_{2a}={\dot{\alpha }}_{1}+a_{1n}x_{2}\\ \alpha_{2s}=\alpha_{2s1}+\alpha_{2s2}\\ \alpha_{2s1}=-k_{2}e_{2}\\ \alpha_{2s2}=-k_{s1}e_{2} \end{array}\right.$$ (22)

In the formula: *k*_2_ represents the linear feedback gain, *k*_*s*1_ represents the nonlinear feedback gain; *α*_2_ comprises two components, where *α*_2*a*_ represents the model compensation, while *α*_2*s*_ represents the robust control rate composed of linear feedback *α*_2*s*1_ and nonlinear feedback *α*_2*s*2_, and *α*_2*s2*_ should satisfy the following conditions:

$$e_{2}[\alpha_{2s2}-D(x,t)]\leq \sigma_{1}$$ (23)

$$e_{2}\alpha_{2s2}\leq 0$$ (24)

In these equations, *σ*_1_ represents a design parameter.

The Lyapunov function is constructed as follows:

$$L_{3}=L_{2}+e_{3}^{2}/2$$ (25)

Differentiating *e*_3_ with respect to time and substituting the result into Eq (10) yields:

$${\dot{e}}_{3}={\dot{x}}_{3}-{\dot{\alpha }}_{2}=a_{2}gu-a_{3}x_{2}-a_{4}x_{3}-a_{5}-{\dot{\alpha }}_{2c}-{\dot{\alpha }}_{2u}$$ (26)

In the formula, ${\dot{\alpha }}_{2}={\dot{\alpha }}_{2c}+{\dot{\alpha }}_{2u},{\dot{\alpha }}_{2c}$ and ${\dot{\alpha }}_{2u}$ represent the computable and uncomputable parts of ${\dot{\alpha }}_{2}$, respectively.

Record $\hat{\dot{x}}=x_{3}-a_{1n}x_{2}$ as the estimation of ${\dot{x}}_{2}$ and ${\tilde{\dot{x}}}_{2}={\hat{\dot{x}}}_{2}-{\dot{x}}_{2}$ as the estimation error of ${\dot{x}}_{2}$. Then ${\dot{\alpha }}_{2c}$ and ${\dot{\alpha }}_{2u}$ are expressed as:

$$\left\{ \begin{array}{c} {\dot{\alpha }}_{2c}=\frac{\partial \alpha_{2}}{\partial t}+(\frac{\partial \alpha_{2}}{\partial x_{1}}){\hat{x}}_{2}+(\frac{\partial \alpha_{2}}{\partial x_{2}}){\hat{\dot{x}}}_{2}\\ {\dot{\alpha }}_{2u}=(\frac{\partial \alpha_{2}}{\partial x_{2}}){\tilde{\dot{x}}}_{2} \end{array}\right.$$ (27)

Differentiating Eq (21) yields:

$${\dot{L}}_{3}=e_{2}[e_{3}+\alpha_{2}-a_{1n}x_{2}-{\dot{\alpha }}_{1}-D(x,t)]+e_{3}(a_{2}gu-a_{3}x_{2}-a_{4}x_{3}-a_{5}-{\dot{\alpha }}_{2c}-{\dot{\alpha }}_{2u})$$ (28)

The robust adaptive controller and adaptive function derived from this analysis are as follows:

$$\left\{ \begin{array}{c} u=\left(u_{a}+u_{s}\right)/g{\hat{a}}_{2}\\ u_{a}={\hat{a}}_{3}x_{2}+{\hat{a}}_{4}x_{3}+{\hat{a}}_{5}+{\dot{\alpha }}_{2c}\\ u_{s}=u_{s1}+u_{s2}\\ u_{s1}=-k_{3}e_{3}\\ u_{s2}=-k_{s2}e_{3}\\ \tau =\varphi e_{3} \end{array}\right.$$ (29)

In the formula: *k*_3_ represents the linear feedback gain, *k*_*s*2_ represents the nonlinear feedback gain; *u* comprises two components, where *u*_*a*_ represents the model compensation, *u*_*s*_ represents the robust control rate composed of linear feedback *u*_*s*1_ and nonlinear feedback *u*_*s*2_, and *u*_*s*2_ should satisfy the following conditions:

$$e_{3}[u_{s2}+{\tilde{a}}^{T}\varphi -{\dot{\alpha }}_{2u}]\leq \sigma_{2}$$ (30)

$$e_{3}u_{s2}\leq 0$$ (31)

In the formula: $\tilde{a}=({\tilde{a}}_{1},{\tilde{a}}_{2},{\tilde{a}}_{3},{\tilde{a}}_{4},{\tilde{a}}_{5});\;\varphi =(0,-gu,x_{2},x_{3},1)$, where *σ*_2_ represents a design parameter.

### 4.3 Design of feedforward compensation based on interference observer

Eq (20) can be reformulated as:

$$\left\{ \begin{array}{c} {\dot{x}}_{1}=x_{2}\\ {\dot{x}}_{2}=x_{3}-a_{1n}x_{2}-D(x,t)\\ {\dot{x}}_{3}=a_{2}gu-a_{3}x_{2}-a_{4}x_{3}-a_{5} \end{array}\right.$$ (32)

The upper bounds of the absolute values of *d*(*x*, *t*) and its first derivative in Eq (10) are denoted as *δ*_1_ and *δ*_2_, respectively; denoted as *a*_2*m*_ = *a*_2*max*_−*a*_2*min*_.

From *D*(*x*, *t*) = (*a*_2_−*a*_2*n*_)*x*_2_−*d*(*x*, *t*), it can be inferred that *D*(*x*, *t*) and its first derivative are bounded, and:

$$\left\{ \begin{array}{c} D(x,t)\leq a_{2m}|x_{2}|+\delta_{1}\\ \dot{D}(x,t)\leq a_{2m}|{\dot{x}}_{2}|+\delta_{2} \end{array}\right.$$ (33)

To estimate the interference *D*(*x*, *t*) in the aforementioned equation. According to reference [35], an interference observer is designed in the following form:

$$\left\{ \begin{array}{c} {\dot{z}}_{0}=v_{0}-a_{2n}x_{2}\\ {\dot{z}}_{1}=v_{1}=\dot{\hat{D}}\\ {\dot{z}}_{2}=v_{2}=\dot{\hat{\dot{D}}}\\ v_{0}=-\lambda_{0}{\left|z_{0}-x_{2}\right|}^{\frac{2}{3}}∙sgn(z_{0}-x_{2})+z_{1}\\ v_{1}=-\lambda_{1}{\left|z_{1}-v_{0}\right|}^{\frac{1}{2}}∙sgn(z_{1}-v_{0})+z_{2}\\ v_{2}=-\lambda_{2}sgn(z_{2}-v_{1}) \end{array}\right.$$ (34)

In the formula, *λ*_*i*_>0, (*i* = 0,1,2) is the adjustable observation coefficient of the disturbance observer, $\hat{D}$ and $\hat{\dot{D}}$ are the estimated values of *D* and $\dot{D}$, respectively.

Lemma: There exists a finite time *T*_1_, when $t>T_{1},\;\tilde{D}=0,\;\tilde{\dot{D}}=0,\;\tilde{D}=\hat{D}-D,\;\tilde{\dot{D}}=\hat{\dot{D}}-D$.

Define saturation function:

$$\left\{ \begin{array}{c} a_{2m}|x_{2}|+\delta_{1},if|\tilde{D}|>a_{2m}|x_{2}|+\delta_{1}\\ \tilde{D},if|\tilde{D}|\leq a_{2m}|x_{2}|+\delta_{1} \end{array}\right.$$ (35)

$$\left\{ \begin{array}{c} a_{2m}|{\dot{x}}_{2}|+\delta_{2},if|\tilde{D}|>a_{2m}|{\dot{x}}_{2}|+\delta_{2}\\ \tilde{\dot{D}},if|\tilde{D}|\leq a_{2m}|{\dot{x}}_{2}|+\delta_{2} \end{array}\right.$$ (36)

From Eq (33) and lemma, it can be concluded that:

$$\left\{ \begin{array}{c} \tilde{D}\leq a_{2m}|x_{2}|+\delta_{1},\exists T_{1},\forall t<T_{1}\\ \tilde{\dot{D}}\leq a_{2m}|{\dot{x}}_{2}|+\delta_{2},\exists T_{1},\forall t<T_{1} \end{array}\right.$$ (37)

By combining Eq (20) with the interference estimation $\hat{D}$, the virtual controller is expressed as:

$$\left\{ \begin{array}{c} \alpha_{2}=\alpha_{2a}+\alpha_{2s}\\ \alpha_{2s}=\alpha_{2s1}+\alpha_{2s2}\\ \alpha_{2a}={\dot{x}}_{2eq}+a_{1n}x_{2}+\hat{D}\\ \alpha_{2s1}=-k_{2}e_{2}\\ \alpha_{2s2}=-k_{s1}e_{2} \end{array}\right.$$ (38)

Substituting Eq (38) into Eq (21) yields:

$${\dot{e}}_{2}=e_{3}-k_{2}e_{2}+\alpha_{2s2}+\tilde{D}$$ (39)

The following properties are exhibited by *α*_2*s*2_:

$$\left\{ \begin{array}{c} e_{2}(\alpha_{2s2}-\tilde{D})\leq \sigma_{3}\\ e_{2}\alpha_{2s2}\leq 0 \end{array}\right.$$ (40)

In the formula, *σ*_3_ denotes a design parameter.

Let *h*_1_ be a smooth curve that satisfies the following conditions:

$$h_{1}\geq a_{2m}|x_{2}|+\delta_{1}$$ (41)

If *h*_1_ is not less than the upper bound of $\tilde{D}$, then *α*_2*s*2_ can be defined as:

$$\alpha_{2s2}=-k_{s1}e_{2}≜-\frac{{e_{2}h}_{1}^{2}}{4\sigma_{3}}$$ (42)

From Eqs (32) and (38), the following expression is derived:

$${\dot{e}}_{3}={\dot{x}}_{3}-{\dot{\alpha }}_{2c}-{\dot{\alpha }}_{2u}=a_{2}gu-a_{3}x_{2}-a_{4}x_{3}-a_{5}-{\dot{\alpha }}_{2c}-{\dot{\alpha }}_{2u}$$ (43)

In the formula, ${\dot{\alpha }}_{2c}$ and ${\dot{\alpha }}_{2u}$ are expressed as:

$$\left\{ \begin{array}{c} {\dot{\alpha }}_{2c}=\frac{\partial \alpha_{2}}{\partial t}+(\frac{\partial \alpha_{2}}{\partial x_{1}}){\hat{x}}_{2}+(\frac{\partial \alpha_{2}}{\partial x_{2}}){\hat{\dot{x}}}_{2}+(\frac{\partial \alpha_{2}}{\partial \hat{D}})\hat{\dot{D}}\\ {\dot{\alpha }}_{2u}=(\frac{\partial \alpha_{2}}{\partial x_{2}}){\tilde{\dot{x}}}_{2} \end{array}\right.$$ (44)

In the formula, ${\hat{\dot{x}}}_{2}$ and ${\tilde{\dot{x}}}_{2}$ are expressed as:

$$\left\{ \begin{array}{c} {\hat{\dot{x}}}_{2}=x_{3}-a_{1n}x_{2}-\hat{D}\\ {\tilde{\dot{x}}}_{2}={\hat{\dot{x}}}_{2}-{\dot{x}}_{2}\\ {\tilde{\dot{x}}}_{2}=-\tilde{D} \end{array}\right.$$ (45)

The robust adaptive controller incorporating interference estimation feedforward compensation is formulated as:

$$\left\{ \begin{array}{c} u=\left(u_{a}+u_{s}\right)/g{\hat{a}}_{2}\\ u_{a}={\hat{a}}_{3}x_{2}+{\hat{a}}_{4}x_{3}+{\hat{a}}_{5}+{\dot{\alpha }}_{2c}\\ u_{s}=u_{s1}+u_{s2}\\ u_{s1}=-k_{3}e_{3}\\ u_{s2}=-k_{s2}e_{3} \end{array}\right.$$ (46)

The nonlinear feedback *u*_*s*2_ must satisfy the following conditions:

$$\left\{ \begin{array}{c} e_{3}[u_{s2}+{\tilde{a}}^{T}\varphi -{\dot{\alpha }}_{2u}]\leq \sigma_{4}\\ e_{3}u_{s2}\leq 0 \end{array}\right.$$ (47)

In the formula, *σ*_2_>0 represents a design parameter.

Let *h*_2_ be a smooth curve that satisfies the following conditions:

$$h_{2}\geq \|\underset{\phi }{\to }\|∙\|\underset{a_{m}}{\to }\|+|\frac{\partial \alpha_{2}}{\partial x_{2}}|∙(a_{2m}|x_{2}|+\delta_{1})$$ (48)

Then *u*_*s*2_ can be defined as:

$$\left\{ \begin{array}{c} u_{s2}=-k_{s2}e_{2}\\ u_{s2}≜-\frac{e_{3}h_{2}^{2}}{4\sigma_{4}} \end{array}\right.$$ (49)

In the formula, *a*_4_>0 denotes a design parameter.

### 4.4 Stability analysis of controller

For the state space Eq (10) of the hydraulic servo position control system of the shock absorber damper test bench, when the controller of Eq (29) is employed as the control input in conjunction with the robust adaptive control rate of Eq (12), the following conclusions can be drawn:

1. All signals within the system are bounded, and the Lyapunov function of the system converges exponentially.
2. In the absence of uncertain nonlinearity (i.e., *D* = 0), the system exhibits asymptotic tracking performance, such that when *t*→∞. *e*_1_, *e*_2_, *e*_3_→0.

The derivative of Eq (25) is expressed as:

$${\dot{L}}_{3}=e_{2}{\dot{e}}_{2}+e_{3}{\dot{e}}_{3}=e_{2}[e_{3}-k_{2}e_{2}+\alpha_{2s2}+D(x,t)]+e_{3}(u_{s2}-k_{3}e_{3}+{\tilde{a}}^{T}\varphi -{\dot{\alpha }}_{2u})\leq e_{2}e_{3}-k_{2}e_{2}^{2}-k_{3}e_{3}^{2}+\sigma_{1}+\sigma_{2}\leq -2\lambda_{min}L_{3}+\sigma_{L}$$ (50)

Where, $\lambda_{min}=min\{k_{1},k_{2}\},\;\sigma_{L}=\sigma_{1}+\sigma_{2}$.

The solution to the differential inequality of Eq (50) is given by:

$$L_{3}\leq e^{-2\lambda_{min}t}L_{3}(0)+\sigma (1-e^{-2\lambda_{min}t})/k$$ (51)

The parameters *e*_1_, *e*_2_, *e*_3_ are globally bounded. When the target displacement is bounded, it can be inferred from the system state space that the system output signal is also bounded. Eq (29) demonstrates that the controller output signal *u* is bounded, thereby confirming conclusion *a*.

In the case where *D* = 0, Eq (50) becomes ${\dot{L}}_{3}\leq -2\lambda_{min}L_{3}$, and after integration, there is $L_{3}\leq e^{-2\lambda_{min}t}L_{3}(0)$. Therefore, conclusion 2 is confirmed.

### 4.5 Design of dung beetle optimization algorithm

**Fig 4:**
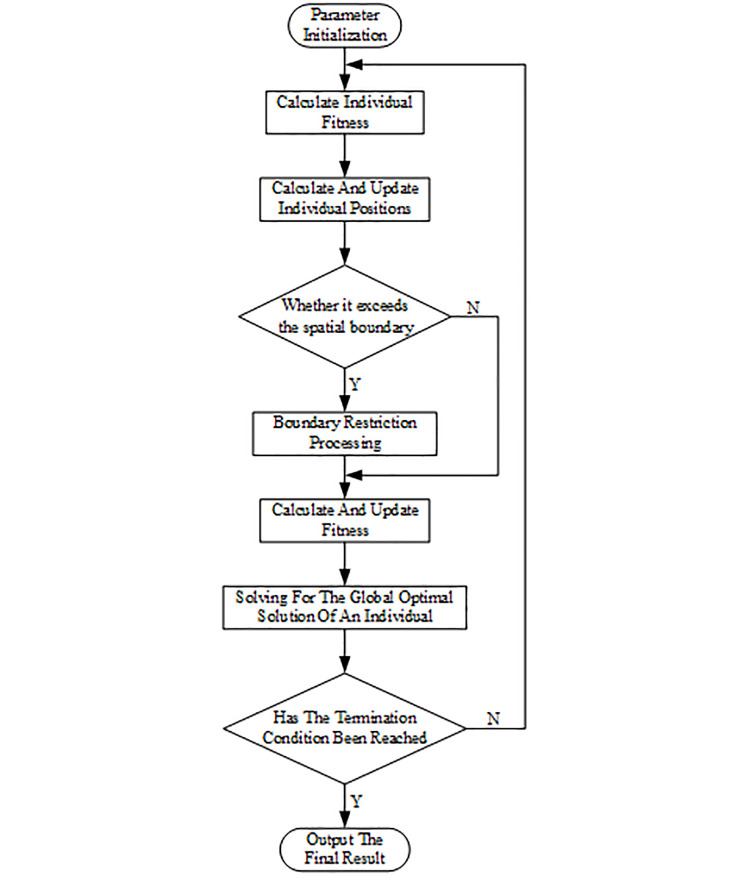
Optimization flowchart of dung beetle optimization algorithm.

The dung beetle optimization algorithm draws inspiration from the behaviors of dung beetles, including rolling dung balls, breeding, foraging, and stealing. These four behaviors correspond to rolling dung beetles, breeding dung beetles, small dung beetles, and stealing dung beetles, respectively. Each type of dung beetle employs distinct position update methods to collectively identify the optimal solution to the problem. The population composition comprises 20% rolling dung beetles, 20% breeding dung beetles, 25% small dung beetles, and 35% stealing dung beetles. Fig 4 illustrates the parameter flow of the dung beetle optimization controller.

#### (1) Rolling ball beetle

In nature, dung beetles continuously roll their feces to form a dung ball. This rolling process requires light for navigation guidance, ensuring the beetle moves along a straight path. However, there exists a probability of encountering obstacles, as described by Eq (52). When rolling the dung ball without encountering obstacles, the beetle’s position is updated according to Eq (53). Upon encountering an obstacle, the beetle must perform a dance on the dung ball to reorient itself and determine a new rolling route. During this reorientation dance, the beetle’s position is updated as shown in Eq (54).


$$\alpha =\left\{ \begin{array}{c} 1,\eta >\lambda \\ -1,\eta <\lambda \end{array}\right.$$ (52)

$$X_{i}(t+1)=X_{i}(t)+\alpha ∙k∙X_{i}(t-1)+b∙\Delta X$$ (53)

$$X_{i}(t+1)=X_{i}(t)+\tan\;\theta |X_{i}(t)-X_{i}(t-1)|$$ (54)

In Eq (52), *η* represents a random number between (0, 1), *λ* represents the probability of the beetle encountering obstacles, and *λ*∈(0, 1). In Eq (53), *k* represents the deviation coefficient, *k*∈(0, 0.02] and *b* represent constants, *b*∈(0, 1) and Δ*X* represent the intensity of light, and Δ*X* = |*X*_*i*_−*X*^*w*^|, where *X*^*w*^ represents the worst position in the population. In Eq (54), *θ*∈[0,*π*] represents the deviation coefficient, and when *θ*∈[0, *π*/2, *π*], tan *θ* becomes meaningless, resulting in no position update.

#### (2) Breeding beetles

**Fig 5:**
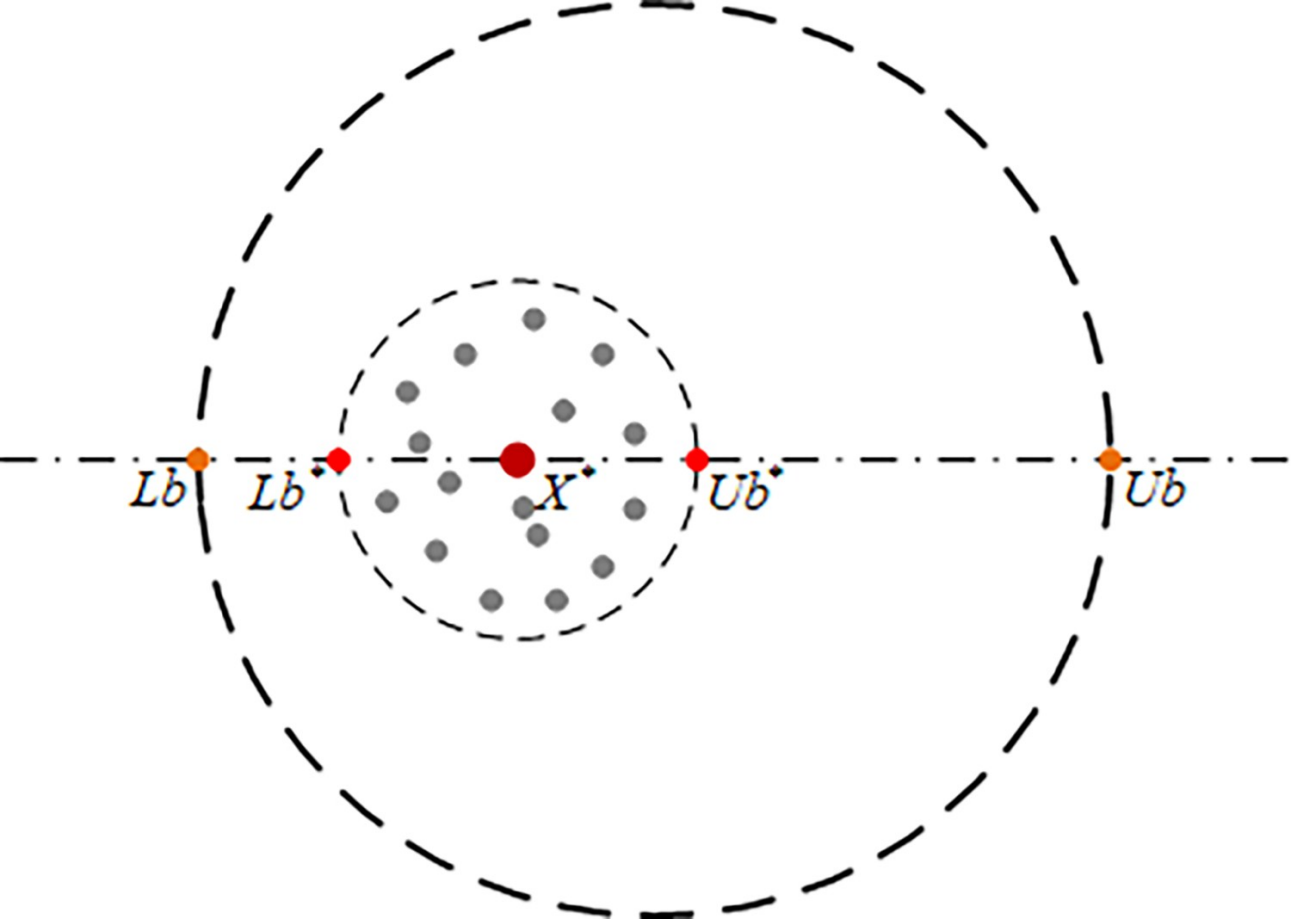
Boundary selection strategy one.

The beetle rolls the dung ball to a secure location and conceals it to provide a safe environment for female dung beetles to lay eggs and protect their offspring, as illustrated in Fig 5. The definition of a safe area is as follows:

$$\left\{ \begin{array}{l} Lb^{*}=max(X^{*}\times (1-R),Lb)\\ Ub^{*}=min(X^{*}\times (1+R),Ub) \end{array}\right.$$ (55)

Where, X* represents the optimal position (local optimal position) *R* = *t*/*T* of the population in this iteration, *Lb* and *Ub* represent the lower and upper bounds of the feasible region, respectively; Ub* Lb* represent the upper and lower bounds of the safe zone, respectively.

Upon identifying a secure area, female beetles proceed to lay eggs within this designated zone. Each female beetle produces a single chick ball, with the position of the chick ball being updated according to Eq (56). In instances where the chick ball’s position exceeds the boundaries of the safe area, Eq (57) is applied.


$$B_{i}(t+1)=X^{*}+b_{1}\times (B_{i}(t)-Lb^{*})+b_{2}\times (B_{i}(t)-Ub^{*})$$ (56)

$$B_{i}=\left\{ \begin{array}{c} Lb^{*},B_{i}<Lb^{*}\\ Ub^{*},B_{i}>Ub^{*} \end{array}\right.$$ (57)

In Eq (56), *b*_1_ and *b*_2_ represent independent random vectors of 1×*W*, while *W* denotes the dimensionality of the optimization problem.

#### (3) Little beetle

**Fig 6:**
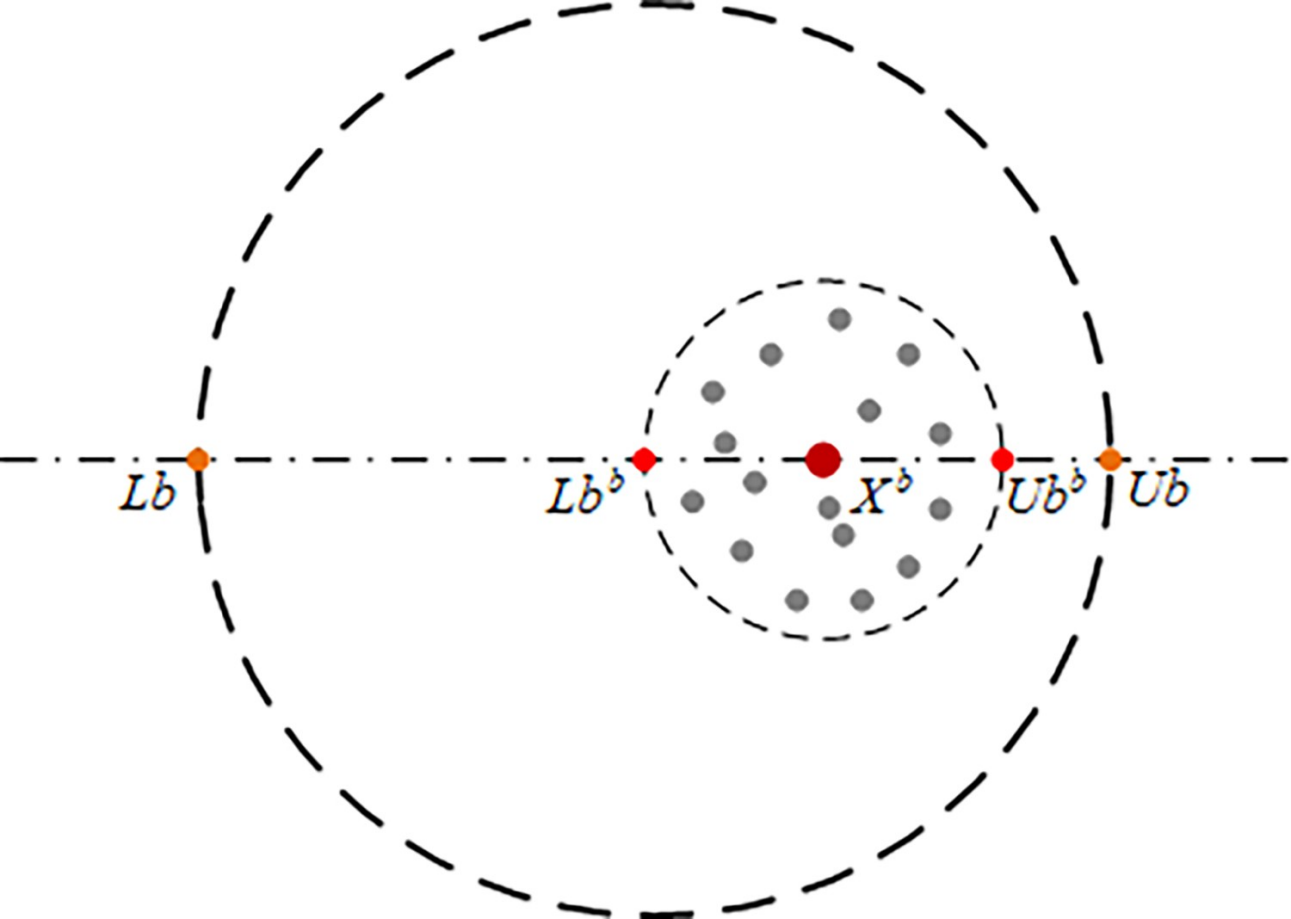
Boundary selection strategy two.

As juvenile beetles mature into small beetles, they emerge from the ground to forage. The foraging locations of small beetles are not arbitrary; instead, these beetles select optimal foraging areas where the probability of finding food is comparatively high, as illustrated in Fig 6. The definition of the optimal foraging area is provided in Eq (58). Once the optimal foraging area has been determined, the position update of the small beetle during foraging is governed by Eq (59):

$$\left\{ \begin{array}{l} Lb^{b}=max(X^{b}\times (1-R),Lb)\\ Ub^{b}=min(X^{b}\times (1+R),Ub) \end{array}\right.$$ (58)

$$X_{i}(t+1)=X_{t}(t)+C_{1}\times (X_{i}(t)-Lb^{b})+C_{2}\times (X_{i}(t)-Ub^{b})$$ (59)

In Eq (58), *X*^*b*^ represents the global optimal position, *Lb*^*b*^ and *Ub*^*b*^ represent the lower and upper bounds of the optimal foraging area, respectively; In Eq (59), *C*_1_ represents a random number vector, where 1×*W* follows a normal distribution, and *C*_2_ is a random vector with values ranging between (0, 1).

#### (4) Stealing beetles

Stealing beetles engage in the theft of dung balls from other beetles. These thefts are not conducted indiscriminately; rather, stealing beetles target the most advantageous locations for theft, which correspond to the global optimal locations. The updated position of stealing beetles is expressed as:

$$X_{i}(t+1)=X^{b}+S\times r\times (\left|X_{i}(t)-X^{*}|+|X_{i}(t)-X^{b}\right|)$$ (60)

In the equation, *r* represents a random vector of size 1×*W* that follows a normal distribution, and *S* represents a constant.

**Fig 7:**
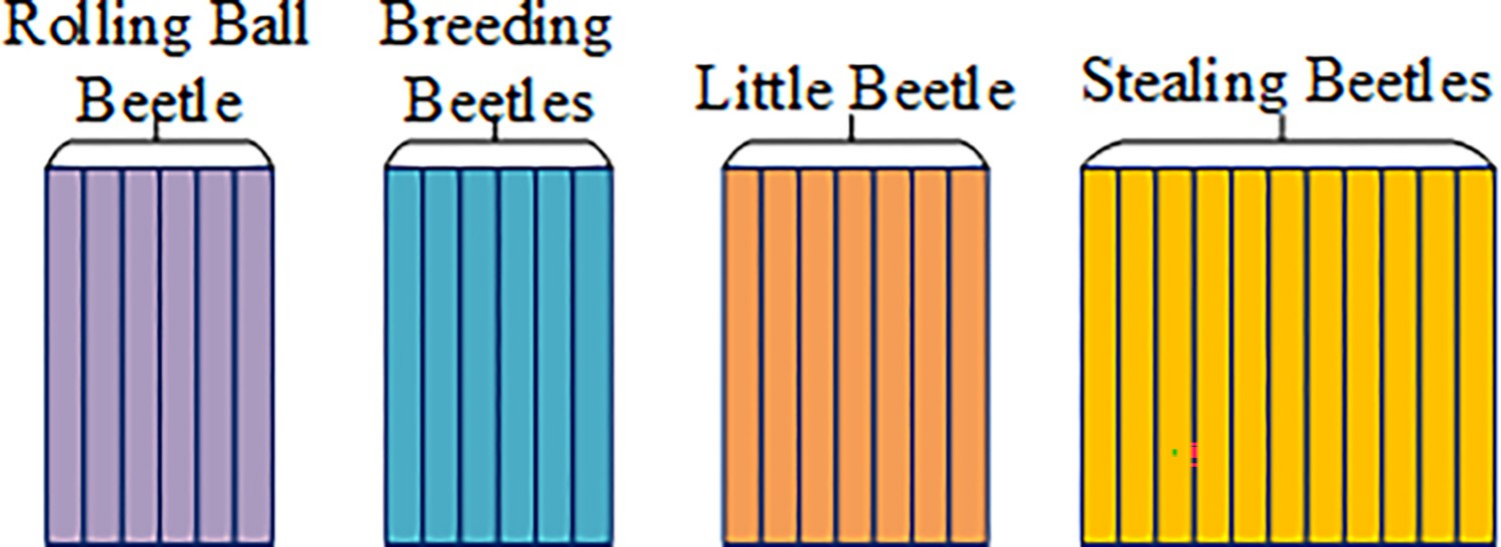
Algorithm DBO search agent distribution.

During the execution of the DBO algorithm, an initial beetle population is randomly generated and allocated across the four aforementioned beetle stages. Through extensive literature review, algorithm designers have gained a deep understanding of the intrinsic relationship between beetle behavior patterns and algorithm performance, and have therefore allocated the number of individuals in each stage reasonably. The individual ratio for each stage is 6:6:7:11, as depicted in Fig 7.

#### (5) Optimal value guidance

In the foraging stage of dung beetles, the generation of candidate solutions is influenced by two random numbers *C*_1_ and *C*_2_. This randomness results in an equal probability of generating both superior and inferior candidate solutions. To enhance the algorithm’s performance, the current optimal value is introduced to guide the generation of candidate solutions. The modified generation formula is expressed as:

$$X_{i}(t+1)=X_{i}(t)+C_{1}(X_{i}(t)-Lb^{b})+C_{2}(X_{i}(t)-Ub^{b})+\lambda \left(X^{b}-X_{i}(t)\right)$$ (61)

In the formula, *λ* represents a random value within the range [0, 1], which is utilized to control the degree of influence exerted by the optimal value. When *λ* is large, the new position is significantly affected by the current optimal position, thereby improving the algorithm’s capacity to exploit the global optimal value discovered in previous iterations.

## 5 Simulation analysis

### 5.1 Model construction

To preliminarily validate the displacement tracking control performance of the proposed DBO-DO-RAC controller, a joint simulation analysis was conducted on the displacement loading system of the shock absorber damper test bench, specifically focusing on the hydraulic servo position control system. This analysis was performed using AEMSim and Simulink software.

**Fig 8:**
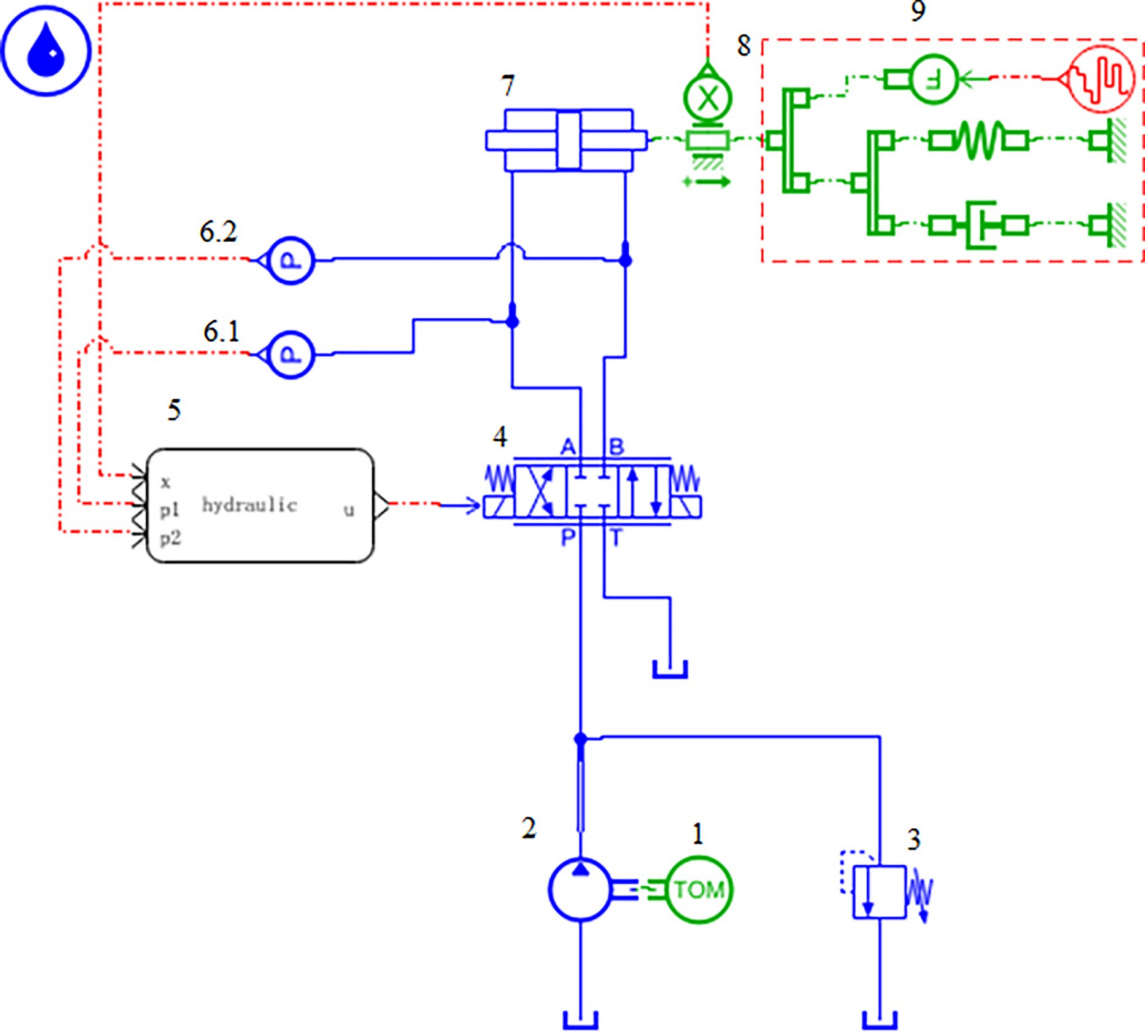
AEMSim model of valve-controlled hydraulic cylinder. 1. Electrical machinery 2. Fixed displacement pump 3. Proportional relief valve 4. Servo Valve 5. Joint simulation interface 6. Pressure sensor 7. Valve-controlled hydraulic cylinder 8. Displacement sensor 9. External load.

As illustrated in Fig 8, a model of the valve-controlled symmetrical hydraulic cylinder system was initially constructed in AEMSim software, and a simulation interface was established. The displacement of the hydraulic cylinder and the pressure of the left and right chambers were output as variables to MATLAB/Simulink. Concurrently, the output signal from Simulink was utilized as the excitation signal for the servo valve to regulate its opening.

**Fig 9:**
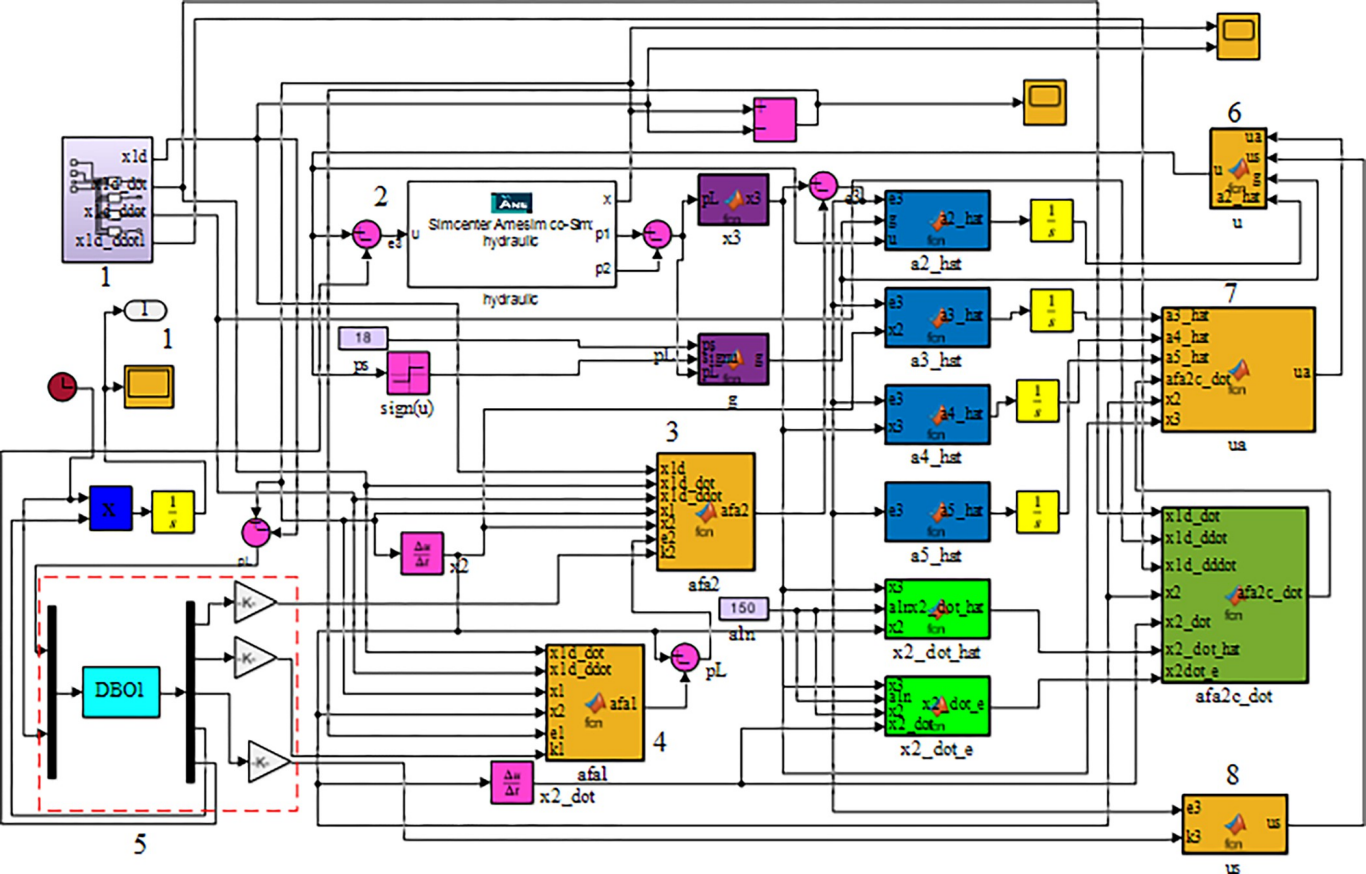
Simulink model of DBO-DO-RAC controller. 1. Target displacement and its derivatives of various orders 2. Joint simulation interface 3.Virtual control rate *α*_2_ 4. Virtual control rate *α*_1_ 5. Optimization parameters of dung beetle optimization algorithm 6. Robust adaptive controller *u* 7. Model compensation item *u*_*a*_ 8. Robust control rate *u*_*s*_.

Subsequently, a control system model of the DBO-DO-RAC controller, as depicted in Fig 9, was developed in MATLAB/Simulink. Necessary oscilloscope modules were incorporated to facilitate data storage in the workspace for subsequent plotting and analysis.

### 5.2 Simulation model parameter settings

The simulation parameters configured in AEMSim and Simulink are presented in Table 1.

**Table 1:** Main parameters of the mode l.

Name/Unit	Symbol	Parameter
Fuel supply pressure/(MPa)	*p_s_*	18
Return oil pressure/(MPa)	*p_r_*	0.07
Hydraulic cylinder inner diameter /(cm)	*D*	40
Diameter of hydraulic cylinder piston rod /(cm)	*d*	20
Hydraulic cylinder stroke/(cm)	*x*	100
Rated current of servo valve/(mA)	*I_n_*	8
Rated flow rate of servo valve /(L/min)	*q_n_*	160
Hydraulic cylinder leakage coefficient /(m^5^/N·s)	*C_ip_*	9.5×10^−12^
Hydraulic cylinder leakage coefficient /(m^5^/N·s)	*C_ep_*	6.5×10^−12^
Equivalent load mass/(kg)	*m_t_*	25
Equivalent spring stiffness /(N·m)	*K*	5×10^4^
Equivalent viscous damping coefficient /(N·s/m)	*B_p_*	1.5×10^5^
Hydraulic oil density /(kg/m^3^)	*ρ*	900
Hydraulic equivalent oil volume modulus of elasticity (Pa)	*β_e_*	7×10^8^

### 5.3 Controller parameter optimization

#### (1) Objective function formulation

During the DBO iteration process, the fitness value of the objective function is employed to determine the superiority or inferiority of individual populations. Consequently, the selection of an appropriate objective function is crucial for accurately reflecting the system state and enhancing control performance. In this study, the time-weighted integral of absolute error (ITAE) is adopted as the objective function, expressed as:

$$ITAE=\int \left[t^{\prime }|e(t^{\prime })|\right]dt^{\prime }=\sum_{i=1}^{n}T(j)|x_{ref}-x(j)|$$ (62)

In Eq (62), *n* represents the sampling point, *T* represents the sampling timing, *e* represents the frequency response curve, *x* represents the error, and *j* represents the *j* sampling point.

#### (2) Parameter optimization process

The optimization process for the DBO-DO-RAC controller involves decoding the initialized population individuals into *k*_1_, *k*_2_ and *k*_3_, and assigning these values to *k*_1_, *k*_2_ and *k*_3_. In the DBO-DO-RAC controller within Simulink. Subsequently, the DBO-DO-RAC controller is simulated, and the objective function ITAE is computed. The algorithm then undergoes iterative updates until the termination condition is satisfied. Upon reaching the termination condition, the optimal solution is output, yielding the optimal control parameters *k*_1_, *k*_2_ and *k*_3_.

#### (3) Analysis of unknown parameter assignmen

Based on the mathematical modeling of the aforementioned electro-hydraulic servo system and the simulation analysis setup, the relevant parameters of the robust adaptive controller are configured as follows: parameter uncertainty range is set to $a_{min}=(0.1,0.1,10^{5},0.1,-10^{5}),\;a_{max}=(500,200,10^{8},100,10^{5})$; parameter estimation initial values are assigned as $a_{1n}=150,\;{\hat{a}}_{2}(0)=25,\;{\hat{a}}_{3}(0)=1\times 10^{6},\;{\hat{a}}_{4}(0)=15,\;{\hat{a}}_{5}(0)=0$.

**Fig 10:**
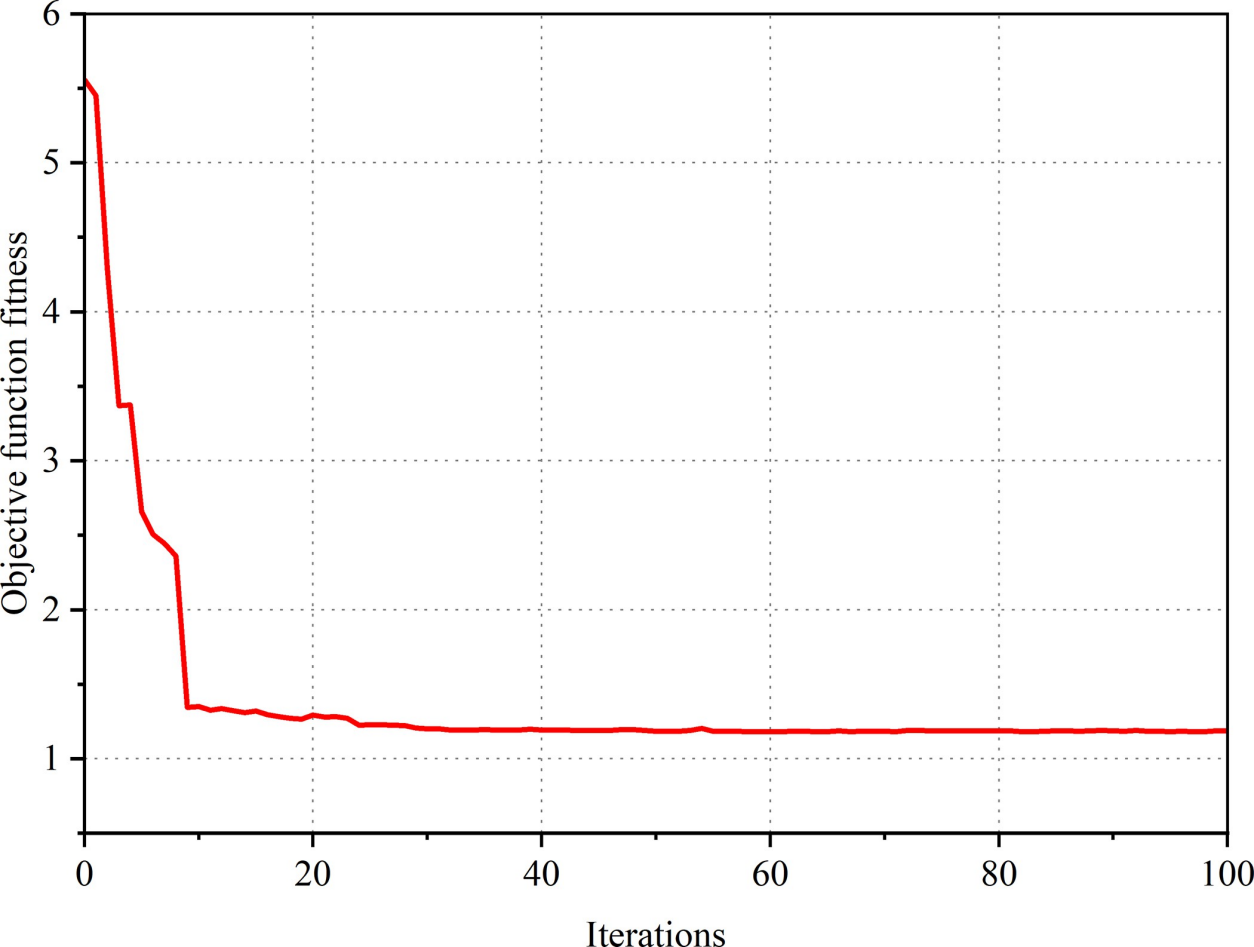
Iterative curve of the dungeon algorithm.

To validate the superiority of the DBO-DO-RAC controller, it was applied to the aforementioned electro-hydraulic servo system for experimentation. The dung beetle optimization algorithm was configured with a population size of 100 and a maximum iteration count of 100. The values of *k*_1_, *k*_2_ and *k*_3_ were constrained within the range of 1 to 2000. The iteration values of the dung beetle optimization algorithm are presented in Fig 10. The resulting control parameters were determined to be *k* = 0.01, *b* = 0.15, *S* = 1.

The optimized control parameters are summarized in Table 2:

**Table 2:** DBO-DO-RAC tuning parameters.

Parameter	DBO-DO-RAC
*k*_1_	1150
*k*_2_	265
*k*_3_	86

### 5.4 Simulation verification

To validate the efficacy of the robust adaptive controller designed in this study, which incorporates the dung beetle optimization algorithm and disturbance observer, a comparative analysis of control performance was conducted. The simulation encompassed the DBO-DO-RAC, RAC, and traditional PID controllers, with the aim of evaluating and contrasting the control performance of these three control strategies

#### (1) Traditional PID controller

The output of a PID controller is expressed as: $u_{PID}=k_{p}(x_{p}-x_{1})+k_{i}\int (x_{p}-x_{1})d(t)+k_{d}\;d(x_{p}-x_{1})/d(t)$, where *k*_*p*_, *k*_*i*_ and *k*_*d*_ represent the proportional coefficient, integral coefficient, and differential coefficient, respectively. The parameters of the PID controller are tuned to the optimal values of *k*_*p*_ = 350, *k*_*i*_ = 70 and *k*_*d*_ = 15 using the Ziegler-Nichols method.

#### (2) Unoptimized robust adaptive controller

The control output of a robust adaptive controller is expressed as: $u=\left(u_{a}+u_{s}\right)/g{\hat{a}}_{2}$, where the control parameters for this controller are identical to those proposed in the article.

### 5.5 Simulation result analysis

In order to quantitatively evaluate the performance of the proposed control method, we used three statistical indicators for performance evaluation, namely the maximum value, average value, and standard deviation of tracking error. Its definition is as follows:

The maximum absolute tracking error is expressed as follows:

$$M_{e}=max\{\left|e(i)\right|\},\;(i=1,\ldots ,N)$$ (63)

In the formula, *N* represents the number of recorded signals.

The average tracking error is expressed as follows:

$$\mu_{e}=\frac{1}{N}\sum_{i=1}^{N}\left|e(i)\right|$$ (64)

The standard deviation of tracking error is expressed as follows:

$$\sigma_{e}=\sqrt{\frac{1}{N}\sum_{i=1}^{N}{\left(|e(i)|-\mu_{e}\right)}^{2}}$$ (65)

This study incorporates a disturbance observer and a dung beetle optimization algorithm to estimate online the uncertainty of external disturbances and internal parameters, respectively. The influence of disturbance and parameter uncertainty on the control system is mitigated through interference compensation and adaptive law implementation. During the simulation process, a nonlinear disturbance *D*(*x*, *t*) was introduced to evaluate system robustness.

**Fig 11:**
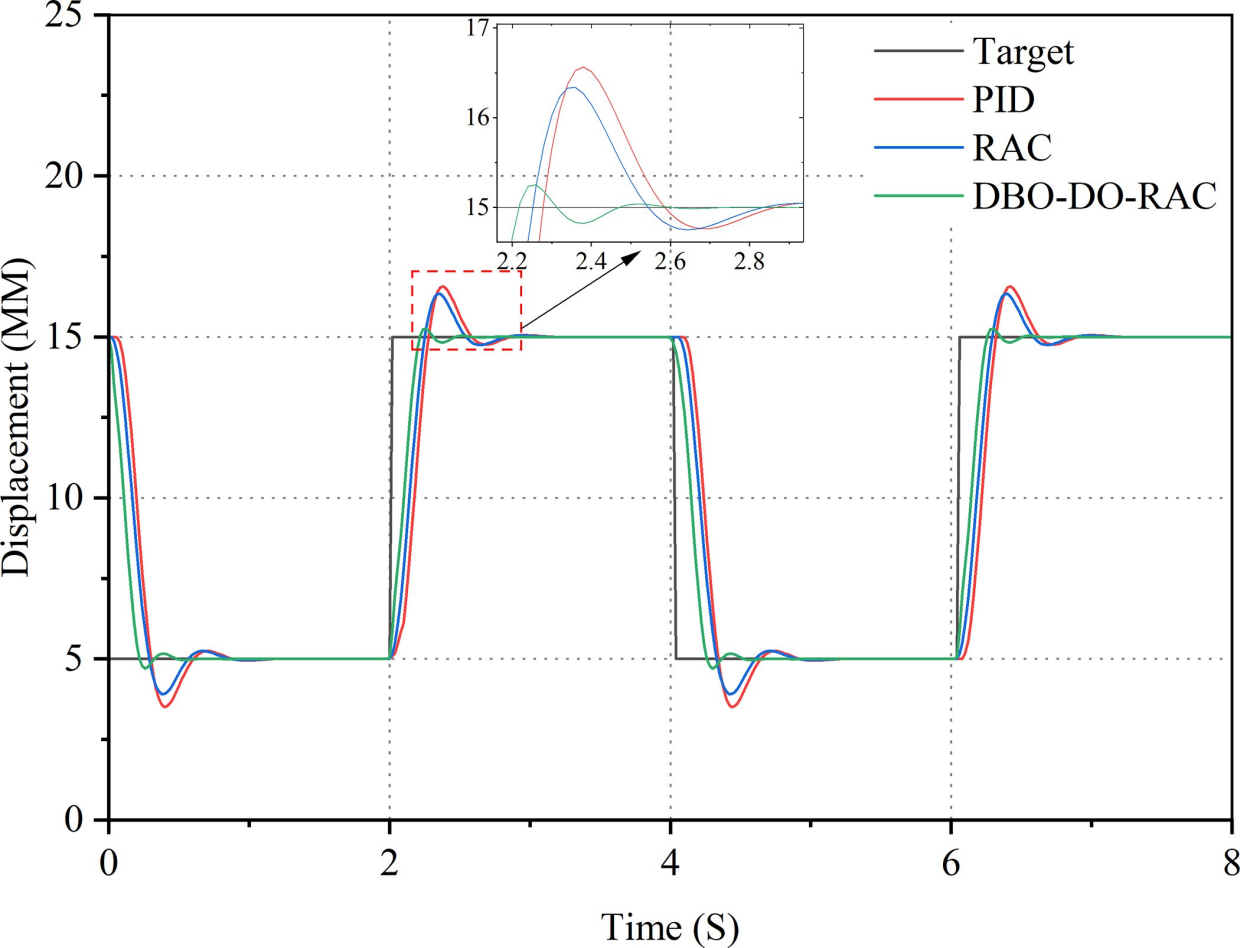
Step signal response curve.

**Fig 12:**
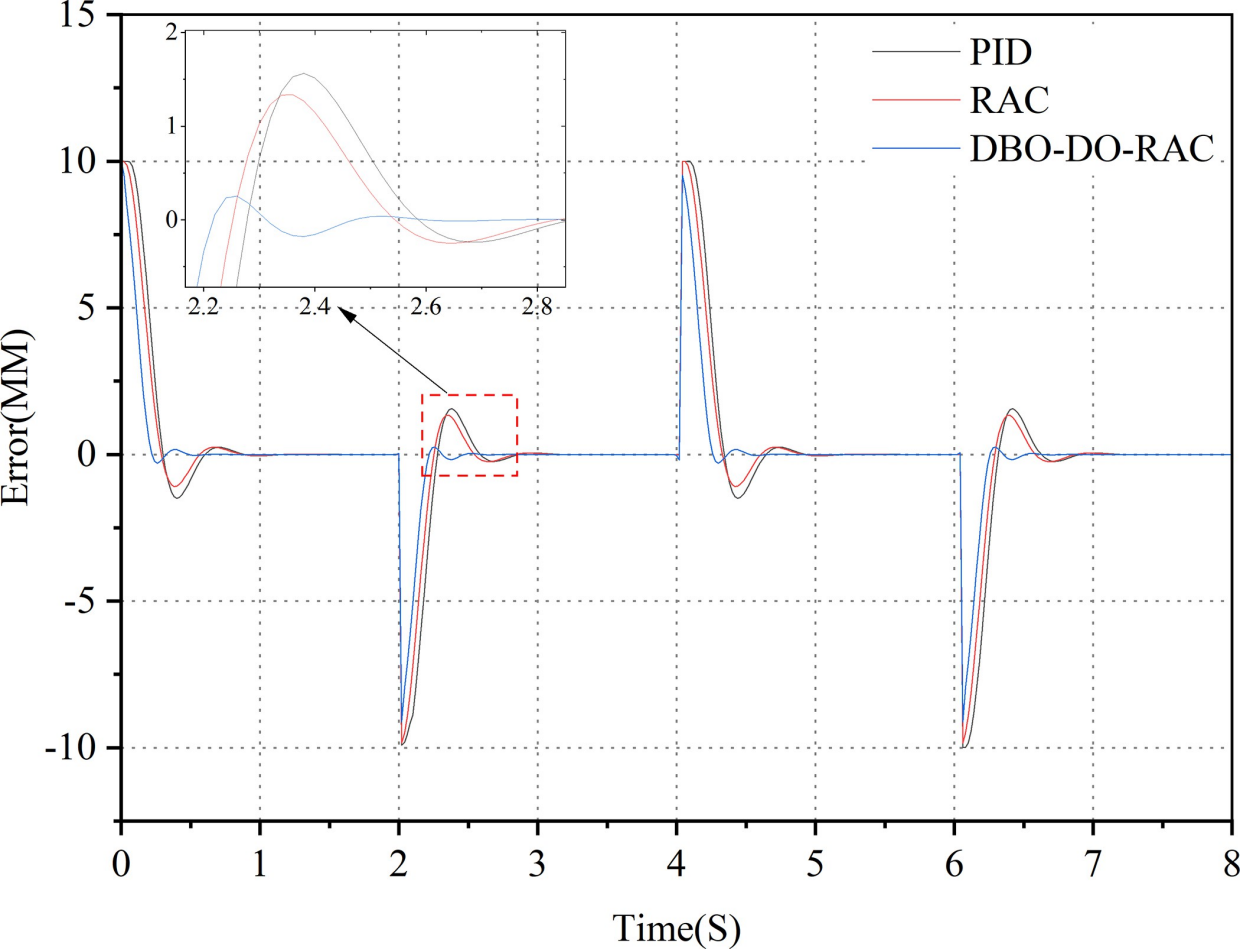
Step signal error curve.

Initially, a step signal was applied as the target input, with an amplitude of 5 mm and a frequency of 0.25 Hz. The response curves of the three control strategies are illustrated in Fig 11, while the corresponding tracking error curves are presented in Fig 12. Table 3 summarizes the performance indicators of the three controllers. Evidently, all three controllers demonstrate an effective response to the target signal in the presence of parameter uncertainty and external interference. Figs 11 and 12 reveal that under these challenging conditions, the DBO-DO-RAC controller exhibits significantly superior performance in terms of overshoot reduction and response speed when compared to the RAC and traditional PID controllers during sudden changes in the step signal. Fig 12 and Table 3 provide quantitative evidence of the DBO-DO-RAC controller’s enhanced performance. The response time of the DBO-DO-RAC controller is reduced by approximately 33.3% compared to the RAC controller and by 37.2% compared to the traditional PID controller, indicating a substantial improvement in response speed. Furthermore, the DBO-DO-RAC controller demonstrates reduced chattering, with a maximum step error reduction of approximately 79.3% compared to the RAC controller and 82.6% compared to the traditional PID controller. Notably, both controllers achieve zero steady-state errors. In summary, the DBO-DO-RAC controller exhibits superior performance characteristics, including smaller transient overshoot error and faster response speed, when compared to its counterparts.

**Table 3:** Comparison of simulation results for step control signals.

Parameter	PID	RAC	DBO -DO- RAC
Maximum step error *M*_*s*_(mm)	1.56	1.31	0.27
Average value *μ*_*e*_(mm)	1.09	0.89	0.53
Standard deviation *σ*_*e*_(mm)	2.56	2.27	1.80
Steady-state time *t*_*s*_(s)	0.51	0.48	0.32
Steady-state error *s*_*e*_(mm)	0	0	0

The control performance indicators of PID controller, RAC controller, and DBO-DO-RAC controller under step signals are detailed in Table 3.

By comparing the performance indexes of the three controllers in Table 3, Under the step signal, it can be found that the average tracking error, the standard deviation of tracking error and the steady-state time of DBO-DO-RAC controller are reduced by 51.3%, 40.4%, 29.7%, 20.7%, and 37.2%, 33.3% compared with PID and RAC controllers. The results show that DBO-DO-RAC controller is superior to the other two controllers in step signal output.

**Fig 13:**
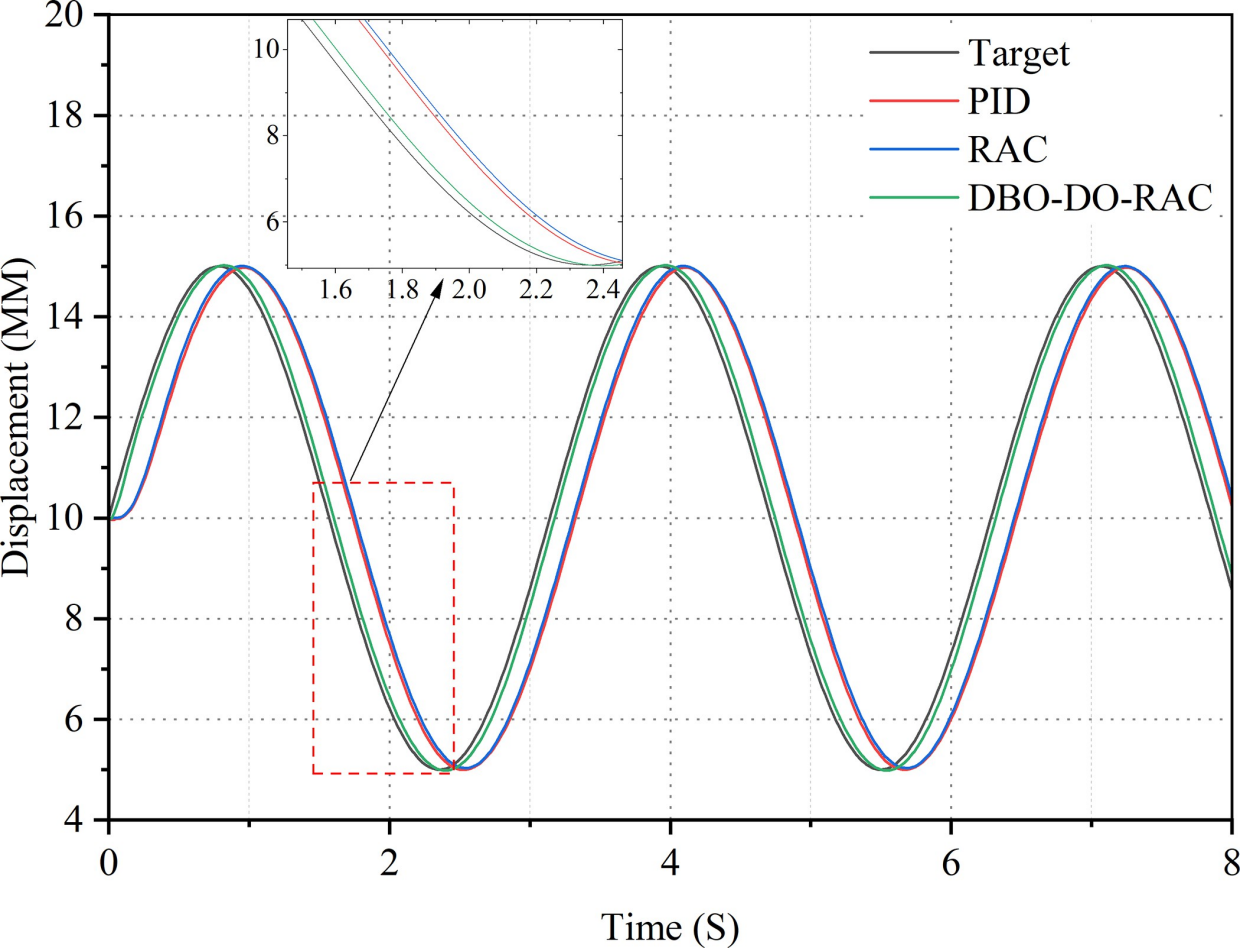
Sinusoidal signal response curve.

**Fig 14:**
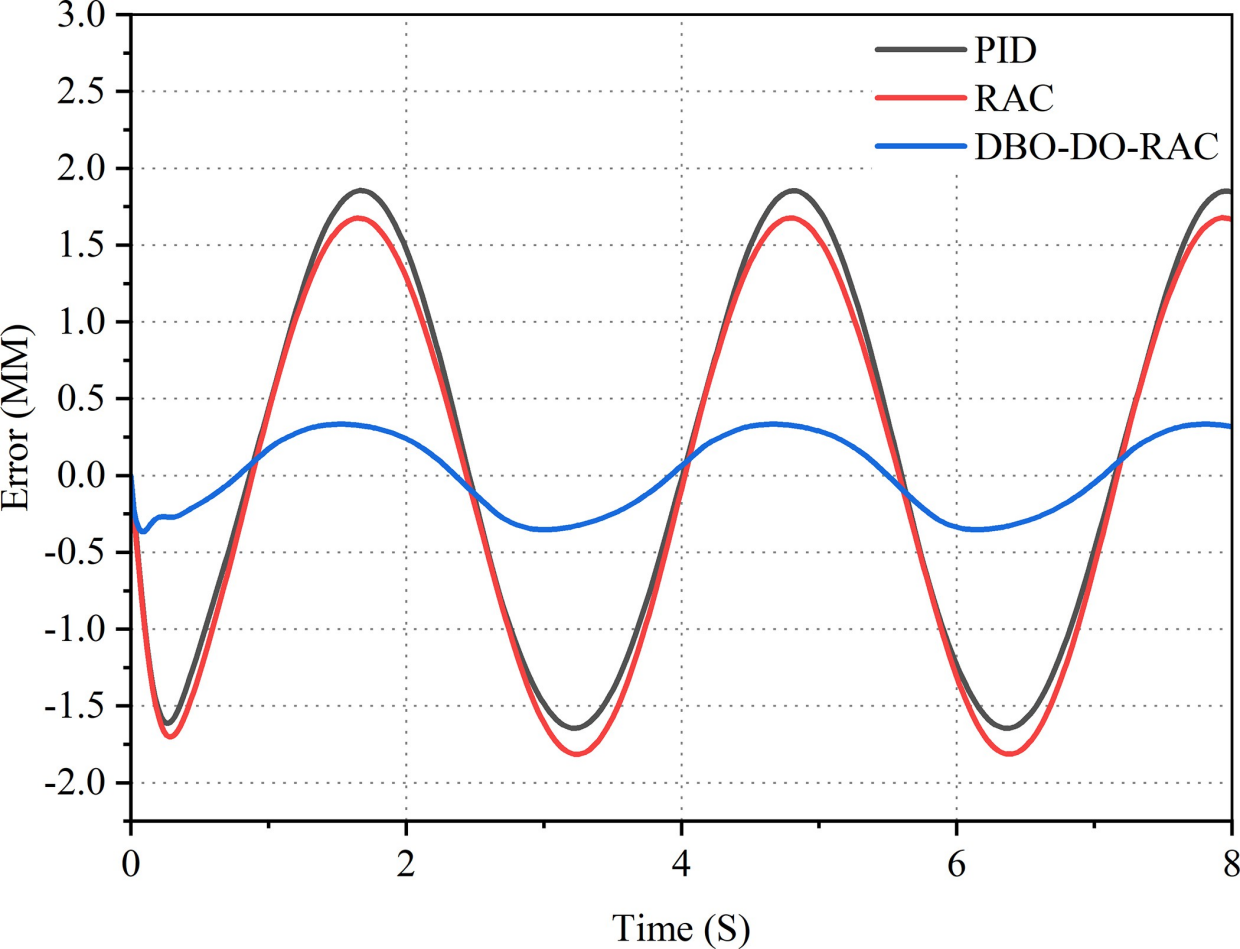
Sinusoidal signal error curve.

Subsequently, a sinusoidal signal was applied as the target input, with an amplitude of 5 mm and a frequency of 0.318 Hz. The displacement response curves for the three control strategies are illustrated in Fig 13, while the corresponding displacement tracking error curves are presented in Fig 14. Evidently, all three controllers demonstrate effective tracking of target signals in the presence of parameter uncertainty and external disturbances. However, the curves in Figs 13 and 14 reveal that under sinusoidal loading signals, the displacement tracking performance of the DBO-DO-RAC controller significantly surpasses that of the RAC and traditional PID controllers. Furthermore, due to external disturbances and parameter uncertainties, the errors of traditional PID controllers and RAC controllers exhibit increased magnitude at the peaks and valleys of the sinusoidal input. The traditional PID controller displays a relatively large displacement tracking error, with a maximum of 1.92 mm. The RAC controller’s maximum displacement tracking error reaches 1.56 mm. In contrast, the DBO-DO-RAC controller maintains a displacement tracking error within 0.35 mm, demonstrating superior tracking performance. Comparative analysis reveals that the DBO-DO-RAC controller reduces the maximum displacement tracking error by approximately 81.8% compared to the traditional PID controller and by about 77.5% compared to the RAC controller. Moreover, the tracking error of the DBO-DO-RAC controller exhibits greater stability, indicating that the integration of the dung beetle optimization algorithm and disturbance observer has significantly enhanced the system’s tracking performance and robustness.

The control performance indicators of PID controller, RAC controller, and DBO-DO-RAC controller under sinusoidal signal are detailed in Table 4.

**Table 4:** Comparison of simulation results for sinusoidal control signals.

Parameter	PID	RAC	DBO -DO- RAC
Maximum absolute error *M*_*e*_(mm)	1.92	1.56	0.35
Average value *μ*_*e*_(mm)	1.13	1.02	0.23
Standard deviation *σ*_*e*_(mm)	0.55	0.53	0.13

By comparing the performance indexes of the three controllers in Table 4, Under the sinusoidal signal, it can be found that the maximum absolute tracking error, average tracking error and standard deviation of tracking error of DBO-DO-RAC controller are reduced by 81.8%, 77.5%, 77.6%, 76.4%, and 79.6%, 75.4% compared with PID and RAC controllers. The results show that the DBO-DO-RAC controller is superior to the other two controllers in sinusoidal signal output.

These results lead to the conclusion that the DBO-DO-RAC controller exhibits excellent tracking performance and transient response characteristics. The maximum tracking error of the system is maintained within 7%, meeting the control performance requirements of the damper test bench. However, it is important to note that many system parameters used in the simulation are theoretical values, introducing a degree of idealization. Consequently, the construction of a physical test bench for displacement loading experiments is necessary to verify the controller’s feasibility in real-world applications.

## 6 Experimental analysis

**Fig 15:**
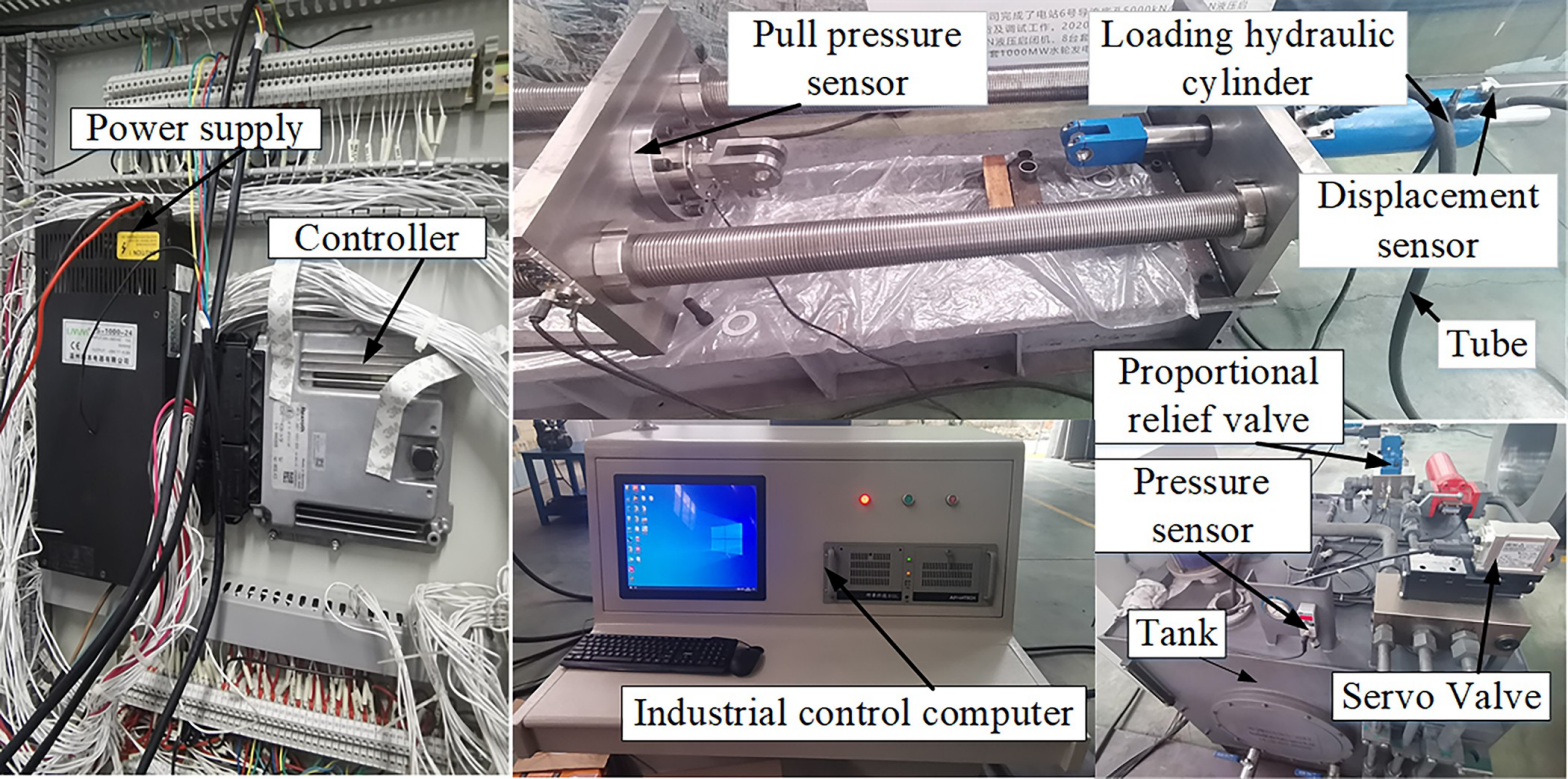
Damping damper test bench.

To validate the actual control performance of the designed DBO-DO-RAC controller in testing damper specimens, displacement tracking control experiments were conducted on a purpose-built damping damper test bench. The constructed shock absorber damper test bench, depicted in Fig 15, comprises four primary subsystems: a displacement loading system, an oil source system, an electronic control system, and an upper computer test control system. The test bench incorporates the following key components: 1. Servo valve: DLKZOR-TEB-SN-NP-L73 (Atos Company). 2. Overflow valve: DBEME10-30/315YG24Z4/A1/2 proportional overflow valve (Lixing Company). 3. Displacement sensor: BTLBNCA00-0500-B15AA010-000S92 external magnetostrictive sensor (Baruff Company), used in conjunction with a 4–20 mA to ±10 V signal isolator (Vidias Company). 4. Force sensor: BKLBNCA00-0500-B15A010-0000S92 external magnetostrictive sensor (China Aerospace Aerodynamics Technology Research Institute). 5. Tension and pressure sensor: BK-4C type. 6. Motion controller: Rexroth control model RC28-14(Rexroth Company). 7. Industrial computer: Advantech’s standard industrial computer.

This article uses the motion RC28-14 as the lower computer and an industrial computer equipped with MATLAB as the upper computer. Implement communication between Simulink and RC28-14 controller through PeakCAN, using Controller Area Network communication. Use Simulink to send valve Controller Area Network information to RC28-14 controller to control valve action, use RC28-14 controller to send sensor Controller Area Network information to Simulink to provide necessary parameters for control algorithm, control sampling period for 2ms, and complete closed-loop tracking control of actual loading displacement and target loading displacement of the oil cylinder.

To validate the efficacy of the DBO-DO-RAC controller on the displacement loading hydraulic cylinder of the shock absorber damper test bench, three experiments were conducted to verify the simulation results of the previous software. The first experiment used a step signal with an amplitude of 5mm and a frequency of 0.5HZ as the target signal. The control strategy proposed in this paper was compared with the traditional PID controller and unoptimized RAC controller that used the Z-N method to optimize the parameters to verify the transient response characteristics. The second experiment uses a sine signal with an amplitude of 5mm and a frequency of 0.425Hz as the target signal to verify the tracking performance of the three control strategies on the target signal. The third one uses a ramp signal with a slope of 0.01 as the target signal to verify the tracking performance of the three control strategies on the target signal under external force interference.

**Fig 16:**
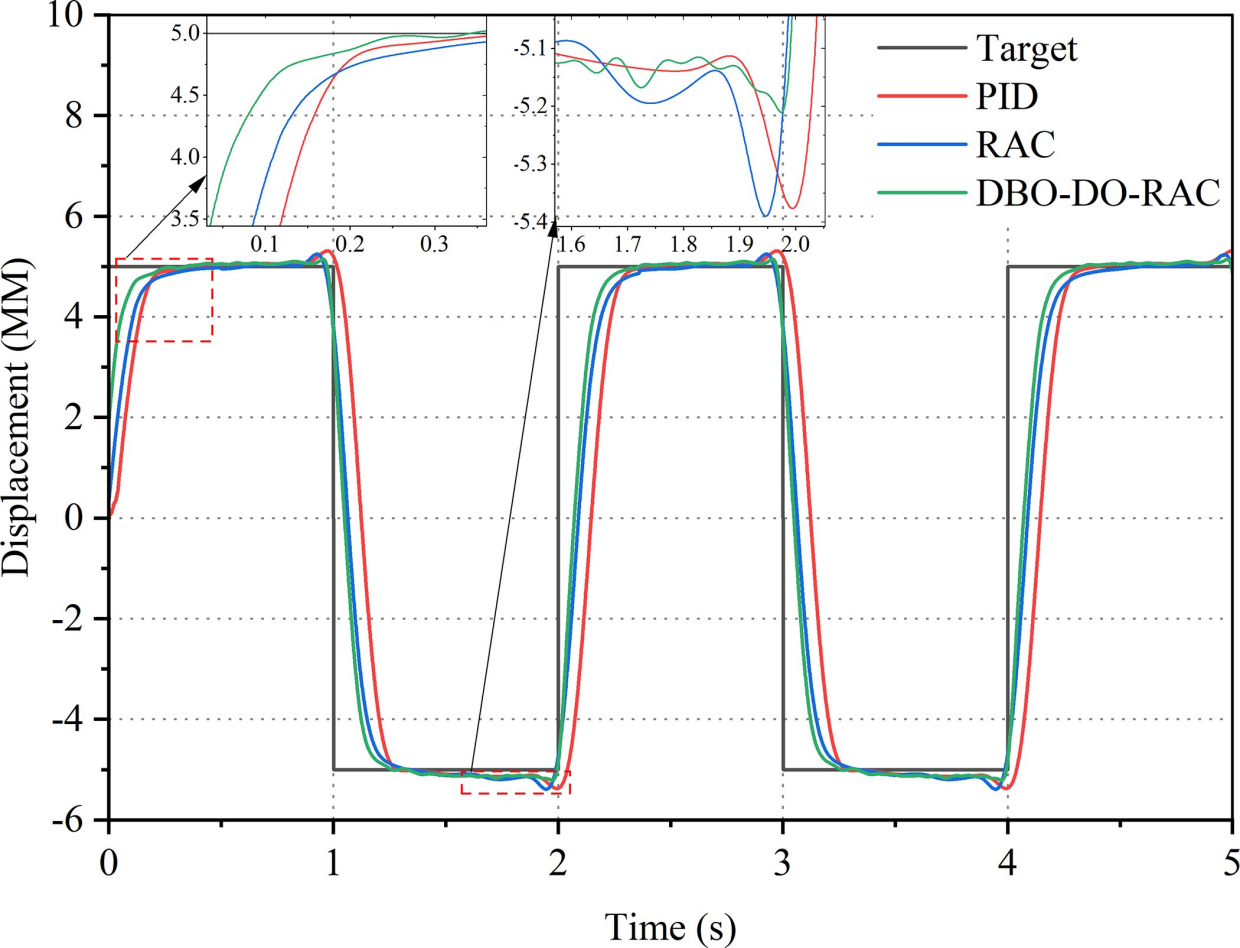
Step signal response curve.

**Fig 17:**
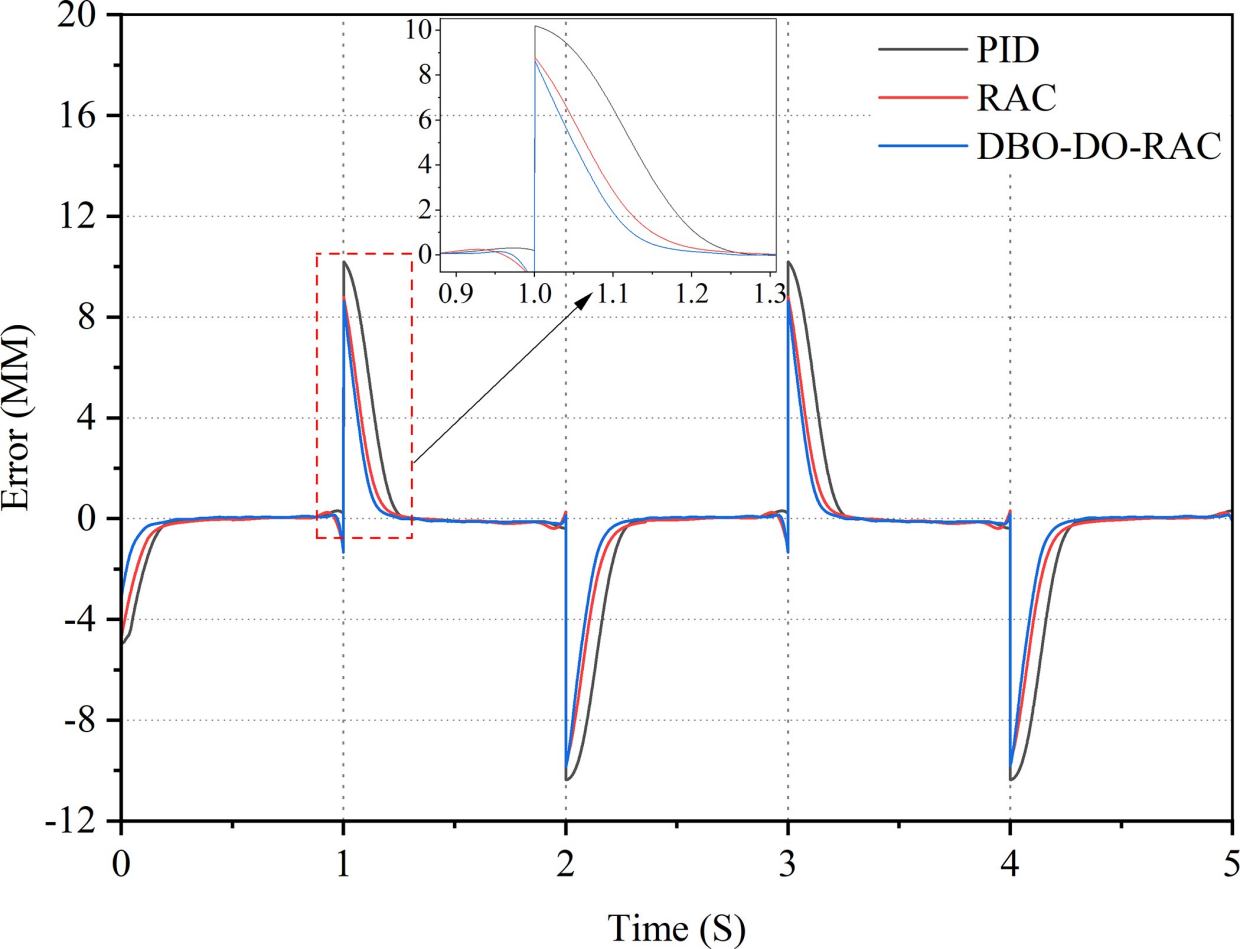
Step signal error curve.

Initially, the transient response characteristics of the DBO-DO-RAC controller were evaluated using a step signal with an amplitude of 5 mm and a period of 0.5 Hz as the target input. Figs 16 and 17 present the displacement response curves and error curves of the hydraulic cylinders controlled by the three methods. These curves reveal significant overshoot in the steady-state phase for both the RAC controller and the traditional PID controller. The DBO-DO-RAC controller demonstrated superior performance with the following characteristics: Rise time of tracking displacement response: approximately 0.35 s; maximum overshoot: approximately 0.1 mm (2% of the target signal); steady-state error: approximately 0.05 mm. In comparison, the RAC controller exhibited: rise time of tracking displacement response: approximately 0.55 s; maximum overshoot: approximately 0.21 mm (4.2% of the target signal); steady-state error: approximately 0.07 mm. The traditional PID controller demonstrated the following: rise time of tracking displacement response: approximately 0.67 s; maximum overshoot: approximately 0.25 mm (5% of the target signal); steady-state error: approximately 0.1 mm. Comparative analysis reveals that the DBO-DO-RAC controller’s response speed increased by approximately 36.3% compared to the RAC controller and by about 47.7% compared to the traditional PID controller. These data demonstrate that the proposed DBO-DO-RAC controller maintains superior tracking performance in the steady-state phase. When compared to the RAC and traditional PID controllers, the DBO-DO-RAC controller exhibits a smaller steady-state error amplitude and enhanced transient response performance.

**Fig 18:**
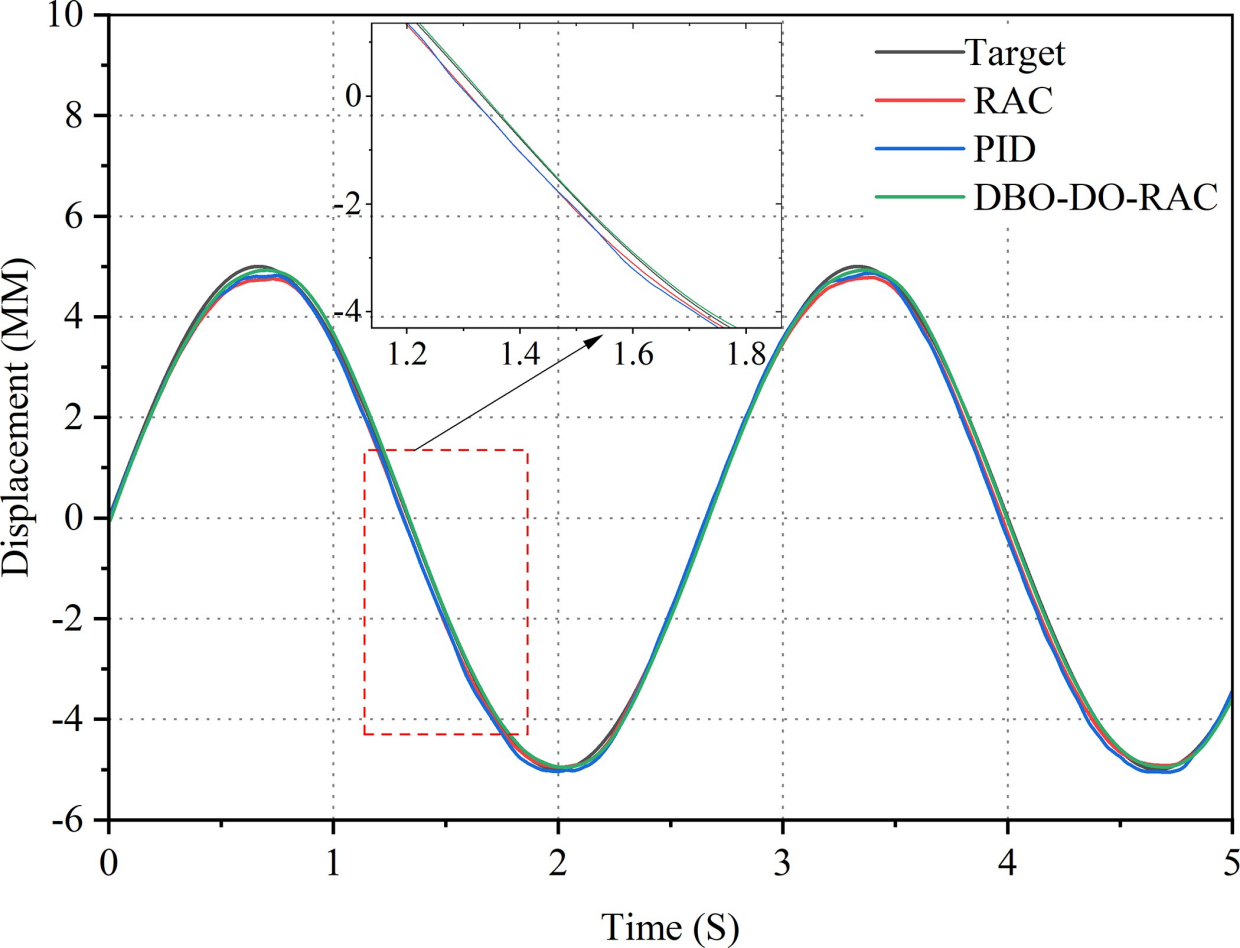
Sinusoidal signal response curve.

**Fig 19:**
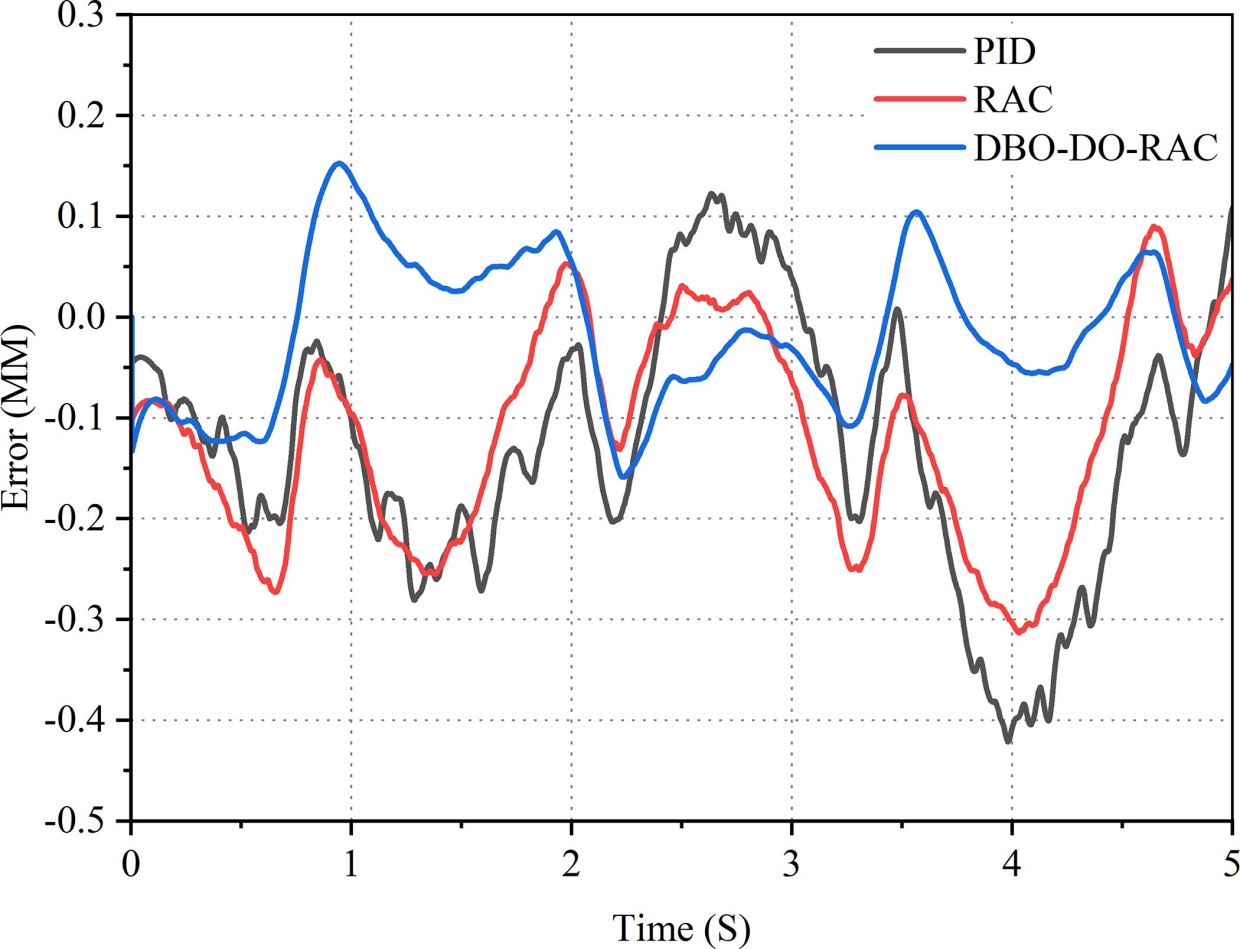
Sinusoidal signal error curve.

Subsequently, the displacement tracking performance of the DBO-DO-RAC controller was evaluated using a sinusoidal signal with an amplitude of 5 mm and a frequency of 0.375 Hz as the target input. The displacement response curves and error curves for the three control strategies are presented in Figs 18 and 19. These figures demonstrate that the tracking curve of the DBO-DO-RAC controller closely approximates the target curve, with relatively small error amplitudes at peak and valley values. The DBO-DO-RAC controller exhibited superior performance with the following characteristics: Maximum displacement tracking error: approximately 0.14 mm; no significant response lead or lag phenomenon observed. In comparison, the RAC controller demonstrated: Maximum displacement tracking error: approximately 0.31 mm; noticeable hysteresis phenomenon. The traditional PID controller showed: Maximum displacement tracking error: approximately 0.43 mm; evident hysteresis phenomenon. Comparative analysis reveals that the DBO-DO-RAC controller reduced the maximum displacement tracking error by approximately 54.8% compared to the RAC controller and by about 67.4% compared to the traditional PID controller. These results indicate that the DBO-DO-RAC controller demonstrates superior adaptability to parameter uncertainties and nonlinear external disturbances, thereby enhancing the controller’s ability to effectively suppress external perturbations. The experimental outcomes corroborate the simulation results, validating the superior performance of the proposed DBO-DO-RAC controller in terms of tracking accuracy, response speed, and disturbance rejection capabilities. The integration of the dung beetle optimization algorithm and disturbance observer significantly enhances the robustness and adaptability of the control system, making it particularly suitable for applications requiring precise displacement control in the presence of uncertainties and disturbances.

**Fig 20:**
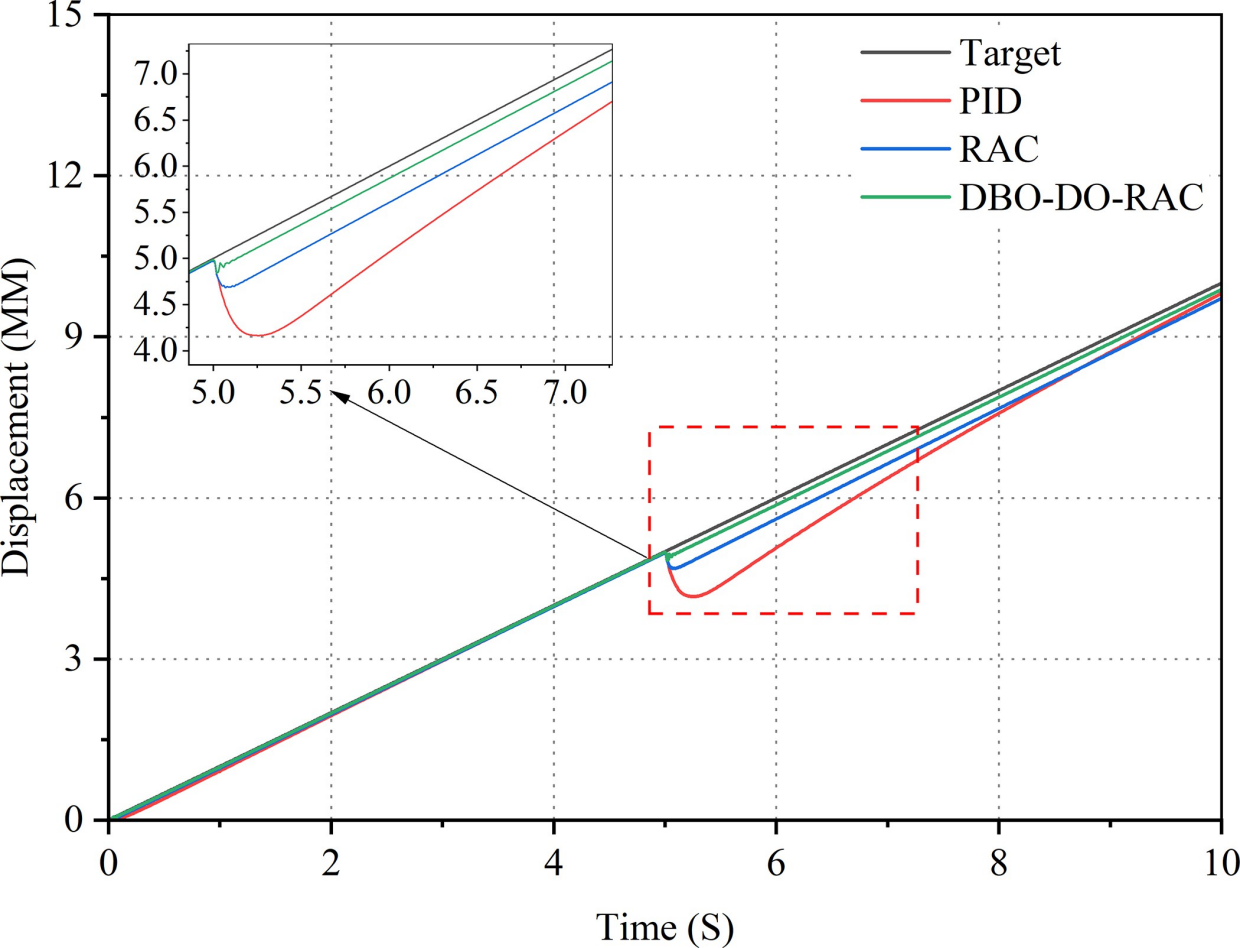
Ramp signal response curve.

**Fig 21:**
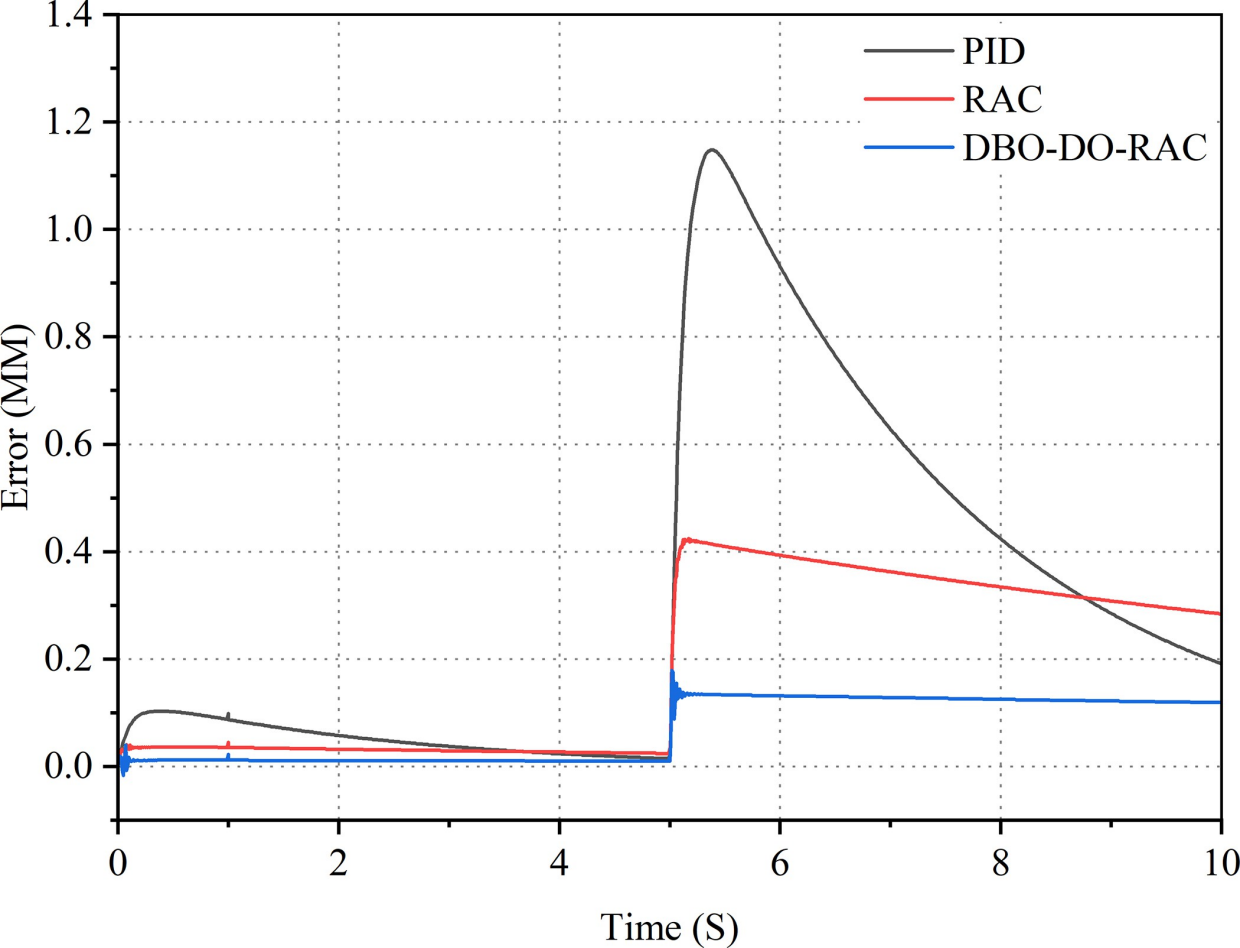
Ramp signal error curve.

Finally, verify the tracking performance of the DBO-DO-RAC controller under external disturbances by setting a ramp signal with a target signal slope of 0.01. The displacement response curves and error curves of the three types of controlled hydraulic cylinders are shown in Figs 20 and 21. From the curves in Figs 20 and 21, it can be seen that the tracking curve of the DBO-DO-RAC controller is less affected by external disturbances. After applying external disturbances during 5S, the disturbance tracking error under the DBO-DO-RAC controller was controlled at 0.18mm and the response was fast; The disturbance tracking error under the RAC controller is 0.43 mm, and the response is slow; The disturbance tracking error under traditional PID controller is 1.15 mm, and the response is significantly delayed. From experimental analysis, it can be concluded that the DBO-DO-RAC controller performs better in adapting to parameter uncertainty and nonlinear external disturbances, and has good performance in suppressing external disturbances.

The control performance indicators of the PID controller, RAC controller, and DBO-DO-RAC controller under the experiment are detailed in Table 5.

**Table 5:** Comparison of experimental signal results.

Signal	Parameter	PID	RAC	DBO-DO-RAC
Step Signal	Steady-state time t_s_(s)	0.67	0.55	0.35
	Average value μ_e_(mm)	0.78	0.70	0.43
	Standard deviation σ_e_(mm)	1.80	1.60	1.05
	Steady-state error s_e_(mm)	0.10	0.07	0.05
Sinusoidal Signal	Maximum absolute error M_e_(mm)	0.43	0.31	0.14
	Average value μ_e_(mm)	0.15	0.13	0.06
	Standard deviation σ_e_(mm)	0.10	0.09	0.05
Ramp Signal	Maximum absolute error M_e_(mm)	1.15	0.43	0.18
	Average value μ_e_(mm)	0.31	0.19	0.07
	Standard deviation σ_e_(mm)	0.33	0.16	0.06

Using the aforementioned data for analysis, statistical analysis was conducted on the three sets of experiments for the DBO-DO-RAC controller with a 95% confidence level. It was assumed that the experimental error data followed a normal distribution, hypothesis H_0_: e = 0, H_1_: e≠0. Firstly, the step signal error was analyzed. Using SPSS software, the confidence interval was found to be [0.4199, 0.4660], with rejection regions of (-∞, -0.5621]∪[0.5621, +∞). The average tracking error for the step signal was 0.443, leading to the conclusion that the null hypothesis H_0_ could not be rejected. Next, statistical analysis was performed on the sinusoidal signal error, resulting in a confidence interval of [0.0653, 0.0667] and rejection regions of (-∞, -0.0862]∪[0.0862, +∞). The average tracking error for the sinusoidal signal was 0.066, also leading to the conclusion that H_0_ could not be rejected. Finally, statistical analysis was conducted on the ramp signal error, yielding a confidence interval of [0.0634, 0.0705] and rejection regions of (-∞, -0.0774]∪[0.0774, +∞). The average tracking error for the ramp signal was 0.067, again leading to the conclusion that H_0_ could not be rejected. Based on this analysis, it can be concluded that the controller proposed in this paper is effective.

By comparing the performance indexes of the three controllers in Table 5, it can be found that under the experimental step signal, the steady-state time, average tracking error and standard deviation of tracking error of DBO-DO-RAC controller are reduced by 47.7%, 36.3%, 44.8%, 38.6%, and 41.7%, 34.4% compared with PID and RAC controllers. Under the experimental sinusoidal signal, it can be found that the maximum tracking error, average tracking error and standard deviation of tracking error of DBO-DO-RAC controller are reduced by 67.4%, 54.8%, 60.0%, 53.8%, and 50.0%, 44.4% compared with PID and RAC controllers. Under the experimental ramp wave signal, it can be found that the maximum tracking error, average tracking error and standard deviation of tracking error of DBO-DO-RAC controller are reduced by 84.3%, 58.1%, 77.4%, 63.2%, and 81.8%, 62.5% compared with PID and RAC controllers. The results show that the DBO-DO-RAC controller is superior to the other two controllers in three kinds of target signal output.

## 7 Conclusion

A robust adaptive control strategy incorporating the dung beetle optimization algorithm and a disturbance observer has been proposed to address the challenges of nonlinearity, parameter uncertainty, and unknown external disturbances in the load displacement tracking of the electro-hydraulic servo control system for the test bench. In the electro-hydraulic servo system, robust adaptive control can effectively compensate the uncertainty and interference of the system, while ensuring the stability and performance of the system. By combining dung beetle algorithm and disturbance observer, the control parameters and compensation strategy can be further optimized, thus improving the robustness and adaptive ability of the system. DBO-DO-RAC is an innovative and effective control strategy. It combines the advantages of intelligent optimization algorithm, control theory and hydraulic technology, and can effectively compensate the uncertainty and interference of the system, while ensuring the stability and performance of the system. This control strategy has a wide application prospect and important research value in electro-mechanical under-actuated systems such as industrial automation, aerospace and robotics.

However, the control method proposed in this article will encounter many challenges when applied to practical engineering. Measures such as continuously optimizing algorithm design, improving controller robustness and adaptability, strengthening system integration and compatibility testing, and establishing effective maintenance and update mechanisms are needed to gradually overcome these challenges and promote the development and application of related technologies.

Then, Matlab/Simulink simulations have demonstrated the superior performance of the proposed control strategy compared to conventional RAC and PID controllers in the presence of parameter uncertainties and unknown external disturbances. The proposed controller exhibited a 33.3% increase in response speed and a 77.5% reduction in displacement tracking error compared to the RAC controller. When compared to traditional PID controllers, the proposed strategy achieved a 37.2% improvement in response speed and a 81.8% reduction in displacement tracking error. These simulation results indicate that the proposed control strategy outperforms both RAC and PID controllers in terms of tracking performance and transient response characteristics across various operating conditions. However, it is acknowledged that the simulation parameters were based on theoretical values, introducing a degree of idealization. Consequently, the construction of a physical test bench for displacement loading experiments was deemed necessary to validate the controller’s practical feasibility.

Finally, the proposed control strategy was subsequently implemented on a damping test bench for experimental verification. Step and sinusoidal input experiments were conducted to evaluate the controller’s performance. The results demonstrated significant improvements in tracking accuracy, response speed, and disturbance rejection capabilities of the electro-hydraulic servo system. Specifically, compared to the RAC controller, the proposed strategy achieved a 36.3% increase in response speed and a 54.8% reduction in displacement tracking error. When benchmarked against traditional PID controllers, the proposed approach exhibited a 47.7% improvement in response speed and a 67.4% reduction in displacement tracking error. These experimental outcomes corroborate the efficacy of the proposed control method in enhancing the tracking accuracy and robustness of the shock absorber damper test bench. The demonstrated improvements in tracking precision, transient response speed, and disturbance rejection capabilities align well with current market demands for advanced electro-hydraulic servo test benches in shock absorber damper applications.

## Supporting information

S1 Data (XLSX)

## Abbreviations

Symbol: Name
*u_i_*: Target displacement signal
*u_d_*: Displacement feedback signal
D: Disturbance
*K_a_*: Amplifier gain
*K_i_*: Displacement sensor gain
*x_v_*: Servo valve spool displacement
*u*: Servo valve input current
*x*_*v*(*max*)_: Maximum displacement of servo valve core
*u_n_*: Rated current of servo valve
*s*: Laplacian
*K_sv_*: Valve core displacement gain
*A_p_*: Hydraulic cylinder with rod chamber compression area
*V_A_*: Effective area of left chamber of hydraulic cylinder
*V_B_*: Effective area of the right chamber of the hydraulic cylinder
*C_ip_*: Leakage coefficient inside hydraulic cylinder
*C_ep_*: External leakage coefficient of hydraulic cylinder
*p_A_*: Left chamber pressure of hydraulic cylinder
*q_A_*: Hydraulic cylinder left chamber flow rate
*p_B_*: Hydraulic cylinder right chamber pressure
*q_B_*: Hydraulic cylinder right chamber flow rate
*m_t_*: Total mass of piston rod
*F_L_*: External load force
*x_p_*: Displacement value of piston rod
*p_s_*: Fuel supply pressure
*p_r_*: Return oil pressure
*p_L_*: Load pressure
*q_L_*: Load traffic
*k_q_*: Flow gain
*C_d_*: Discharge coefficient
ω: Gradient of servo valve slide area
*ρ*: Hydraulic oil density
*k_l_*: System traffic gain
*β_e_*: Equivalent bulk modulus of elasticity of hydraulic oil
*q_n_*: Constant modeling error
*V_t_*: Total force capacity of hydraulic cylinder
*C_ip_*: Total leakage coefficient of hydraulic cylinder
*f*: Unmodeled disturbance
*B_p_*: Viscous damping coefficient
*a*: Unknown parameter set
Γ: Adaptive rate diagonal matrix
*τ*: Adaptation function
${Proj}_{\hat{a}}({}^{\circ})$: Discrete projection function
*x*_1*d*_: Target displacement
*α*_1_: Virtual control rate
*α*_2_: Virtual control rate
*k*_1_: Feedback gain
*k*_2_: Linear feedback gain
*k*_*s*1_: Nonlinear feedback gain
*α*_2*a*_: Virtual model compensation
*α*_2*s*_: Virtual robust control rate
*α*_2*s*1_: Virtual linear feedback
*α*_2*s*2_: Virtual nonlinear feedback
*α*_1_: Design parameters
*k*_3_: Linear feedback gain
*k*_*s*2_: Nonlinear feedback gain
*u_a_*: Model compensation
*u_s_*: Robust control rate
*u*_*s*1_: Linear feedback
*u*_*s*2_: Nonlinear feedback
*σ*_2_: Design parameters
*λ_i_*: Observation parameters
*Q*: Time
*h*_2_: Function curve
*σ*_4_: Design parameters
*η*: Random number
*λ*: Probability of encountering obstacles
*k*: Deviation coefficient
*b*: Constant
*ΔX*: Intensity of light
*X^w^*: The worst position of the population
*θ*: Deviation coefficient
*X**: The optimal position for the group
*Lb*: Lower bound of feasible region
*Ub*: Upper bound of feasible area
*Ub**: Upper bound of safe zone
*Lb**: Lower bound of safe zone
*b*_1_: Independent vector
*b*_2_: Independent vector
*W*: Optimize dimension
*X^b^*: Global optimal position
*Lb^b^*: Lower bound of optimal foraging area
*Ub^b^*: Upper bound of optimal foraging area
*C*_1_: Random number vector
*C*_2_: Random vector with a range of (0.1)
*r*: Random vector
*S*: Constant
*n*: Sampling point
*T*: Sampling timing
*x*: The frequency response
*e*: Error
*j*: Sampling point
*M_s_*: Maximum step error
*M_e_*: Maximum absolute value error
*μ_e_*: Average value
*σ_e_*: Standard deviation
*s_e_*: Steady-state error
*t_s_*: Steady-state time
